# The Effects of Inspiratory Muscle Training in Critically ill Adults: A Systematic Review and Meta-Analysis

**DOI:** 10.1177/08850666251317473

**Published:** 2025-02-09

**Authors:** Christopher Farley, Ana Oliveira, Dina Brooks, Anastasia N. L. Newman

**Affiliations:** 1 Faculty of Health Science, School of Rehabilitation Science, 3710 McMaster University, Hamilton, ON, Canada; 2 Lab 3R – Respiratory Research and Rehabilitation Laboratory, School of Health Sciences (ESSUA) and Institute of Biomedicine, Aveiro, Portugal; 3 Department of Respiratory Medicine, 27375 West Park Healthcare Centre, Toronto, ON, Canada

**Keywords:** rehabilitation, critical illness, critical care, intensive care units, resistance training, breathing exercises, respiration, artificial

## Abstract

**Purpose:**

The onset of diaphragmatic weakness begins within hours of commencing invasive mechanical ventilation (IMV), which may contribute to the physical disability that can persist at five years after intensive care unit (ICU) discharge. Inspiratory muscle training (IMT) has the potential to alleviate the negative effects of IMV.

**Methods:**

We conducted a systematic review and meta-analysis with an approach consistent with Cochrane methods. We registered our review a priori (PROSPERO: CRD 42023451809) and published our protocol. Randomized controlled trials (RCTs) which enrolled adults (≥18 years) admitted to ICU who required IMV for ≥24 h were eligible if they delivered an IMT intervention using an external device that provided airway resistance (eg, threshold device, tapered flow resistive device) compared to usual care. Our primary outcome was physical function. Secondary outcomes included respiratory muscle strength, mortality, length of stay, IMV weaning time, reintubation rate, dyspnea and endurance. We searched Medline, Embase, Emcare, AMED, CINAHL, CENTRAL and clinicaltrials.gov from inception and used the Covidence platform for study selection and data extraction. We reported results as standardized mean difference (SMD) if outcome measures were similar. We used the Grading of Recommendations, Assessment, Development and Evaluations (GRADE) to assess the certainty of evidence.

**Results:**

We screened 12 945 studies and 18 met the inclusion criteria. Three studies reported the effects of IMT on physical function. IMT may have no effect on physical function (SMD = −0.05, 95% confidence interval: −0.46 to 0.36) however results are very uncertain.

**Conclusion:**

Our results suggest physical function is not impacted by IMT; however, our results are based on a limited number of studies with small samples sizes. High quality, appropriately powered RCTs are needed to improve the precision of the effect estimate.

## Introduction

Diaphragmatic atrophy develops within hours of initiating invasive mechanical ventilation (IMV) in the intensive care unit (ICU).^1^ This results in diaphragmatic weakness, which is defined as a decrease in the force producing capacity of the diaphragm.^2^ Respiratory muscle weakness at extubation, defined as a maximal inspiratory pressure (MIP) score of ≤ 30 cmH_2_O, has been associated with a one-year mortality of 31%; conversely, those with a MIP score above 30 cmH_2_O had a 7% one-year mortality.^3^ This respiratory muscle weakness may also contribute to the physical disability that persists in survivors of critical illness 5 years after discharge.^4^

Inspiratory muscle training (IMT) is an intervention that has the potential to improve outcomes for patients who have required IMV. Similar to skeletal muscle strength training, IMT involves the targeted application of an external load to the respiratory muscles during the inspiratory phase of breathing with the goal of improving muscle strength and endurance.^5^ A previous review showed that IMT in people with critical illness may increase inspiratory muscle strength, and reduce IMV weaning time, ICU length of stay and mortality; however, the certainty of evidence for these outcomes was either low or very low.^5^

Despite the positive effects IMT may have on some outcomes, to our knowledge, no review has yet synthesized the effects of IMT on physical function outcomes. Systematic reviews and meta-analyses have demonstrated the positive effect of IMT on physical function in people with chronic obstructive pulmonary disease,^6^ COVID-19,^7^ heart failure,^8^ and obesity.^9^ As such, we aimed to answer the following question: In adults admitted to the ICU who required IMV for ≥24 h, does IMT using an external device that provided airway resistance compared to usual care improve physical function outcomes?

## Methods

This review was registered a priori with the International Prospective Register of Systematic Reviews (PROSPERO) (CRD 42023451809). It was reported according to the Preferred Reporting Items for Systematic reviews and Meta-Analyses (PRISMA) 2020 statement (eTable 1),^10^ and was conducted with methods consistent with the Cochrane Handbook for Systematic Reviews of Interventions.^11^ The methods were outlined in our published protocol,^12^ and are described below.

### Eligibility Criteria

Randomized controlled trials (RCTs) were eligible for inclusion if they enrolled adults (≥18 years) admitted to the ICU who required ≥24 h of IMV. Our protocol stated our intent to only include interventions which used a threshold device.^12^ Due to inconsistent reporting of IMT device type, we modified our protocol to include any IMT intervention which used an external device that provided airway resistance (eg, threshold device, tapered flow resistive device) with a goal of improving inspiratory muscle strength compared to usual care. We had intended to operationalize the goal of improving respiratory muscle strength by only including studies which assessed MIP; we eliminated this criterion due to the potential of excluding otherwise eligible studies that had assessed our primary outcome. Our primary outcome was physical function with secondary outcomes including mortality, length of stay (hospital and ICU), IMV weaning time, reintubation rate, dyspnea, MIP, maximal expiratory pressure (MEP) and respiratory endurance. Studies were included if they were reported in English, French or Portuguese.

### Information Sources

We searched MEDLINE, Embase, Emcare, AMED, CINAHL, and CENTRAL from database inception to April 13, 2024. Similarly, we searched ClinicalTrials.gov to April 13, 2024, and reviewed reference lists of included studies for relevant publications.

### Search Strategy

Search strategies were developed in consultation with a health sciences librarian and included three search concepts: (i) critical illness, (ii) inspiratory muscle training, and (iii) randomized controlled trial (eTables 2-8).

### Selection Process

Citations identified from the search were imported into Covidence systematic review software (2020, Veritas Health Innovation, Melbourne, VIC, Australia), which automatically removed duplicates. To ensure reviewer consistency, we completed a calibration exercise of 10 studies prior to title/abstract screening and 5 studies prior to full text screening. Both title/abstract and full text screening were manually completed independently and in duplicate using the Covidence platform. Disagreements were resolved with discussion and, if needed, by a third reviewer.

### Data Collection Process

Data extraction was manually completed independently and in duplicate using the Covidence platform. If outcomes were reported such that they could not be included in a meta-analysis, we attempted to contact study authors by email for clarification. A third reviewer was available to arbitrate disagreements, if needed.

### Data Items

Extracted data included study identification details (eg, first author, funding sources), participant characteristics (eg, eligibility criteria), intervention and comparator group characteristics (eg, treatment description), and outcomes assessed (eg, outcome measures, assessment timepoints and results). Our protocol indicated our plan to extract outcome assessments at the ICU discharge, hospital discharge and 3-, 6-, and 12-month post discharge timepoints; we also indicated the potential need to refine timepoints based on the included studies.^12^

### Risk of Bias Assessment

Risk of bias was manually assessed using the Cochrane tool for assessing risk of bias in randomized trials version 2 (RoB 2)^13^ independently and in duplicate, with a third reviewer to achieve consensus, as needed. The RoB 2 includes 5 domains of potential bias: the randomization process, deviation from the intervention interventions, missing outcome data, outcome measurement, and selective reporting. Each domain was assessed as either low risk of bias, some concerns or high risk of bias per outcome extracted.

### Data Analysis and Synthesis

Descriptive statistics were used to summarize the study characteristics. Continuous data were reported as mean (standard deviation [SD]) or median (first-third quartiles [Q]), as appropriate. Nominal and categorical variables were summarized as counts and percentages.

#### Effect Measures

Continuous data were reported as mean difference (MD) if the outcome measures used were consistent or standardized mean difference (SMD) if outcome measures were similar but different. Effect sizes were interpreted according to Cohen's d: small = 0.2; medium = 0.5; and large = 0.8.^14^ Dichotomous data were summarized using risk ratios. All effect measures were reported with 95% confidence intervals (CI).

#### Assessment of Heterogeneity

Clinical heterogeneity was assessed in consideration of the characteristics of the study sample, intervention and comparator characteristics and the mode of outcome assessments. Statistical heterogeneity was assessed with visual inspection of forest plots, and by considering the Chi^2^ test and the I^2^ statistic.

#### Meta-Analyses

If extracted results were sufficiently homogeneous, we conducted a meta-analysis. In anticipation that interventions would not be identical across studies, we employed a random-effects model using the DerSimonian and Laird method. Forest plots were created to depict all meta-analyses. Meta-analyses were completed using RevMan Web (Version: 7.9.0). Where data from a study could not be included in a meta-analysis due to the absence of necessary data, we summarized the results narratively.

#### Subgroup Analyses

Planned study-level subgroup analyses included (i) time of IMT initiation; (ii) duration of IMV; (iii) treatment fidelity; (iv) diagnosis; and (v) age. In our protocol, we identified our intent to assess duration of IMV by comparing prolonged IMV (≥96 h) to short-term IMV (<96 h). Since the time of IMT initiation within the included studies was so inconsistent with our a priori threshold, we modified the subgroups by comparing prolonged IMV (≥7 days) to short-term IMV (<7 days). We defined treatment fidelity as the extent to which the intervention occurred as intended.^15^

#### Assessment of Reporting Bias

Reporting bias was assessed with the RoB 2 selective reporting domain by comparing the reported results with the associated study protocol or trial registration, if available. For each outcome that was reported in at least 10 studies, we assessed publication bias by visual inspection of a funnel plot.

### Certainty Assessment

The certainty of evidence was assessed for each outcome using the Grading of Recommendations, Assessment, Development and Evaluations (GRADE) tool.^16^ GRADE was used to evaluate the quality of evidence by considering risk of bias, inconsistency, indirectness, imprecision and publication bias. Assessments were completed independently and in duplicate with a third reviewer to arbitrate as needed. Results of GRADE evaluations were reported both narratively according to published guidelines^17^ and in a summary of findings table, which was created using GRADEpro GDT (McMaster University and Evidence Prime, 2024).

## Results

**Figure 1.**
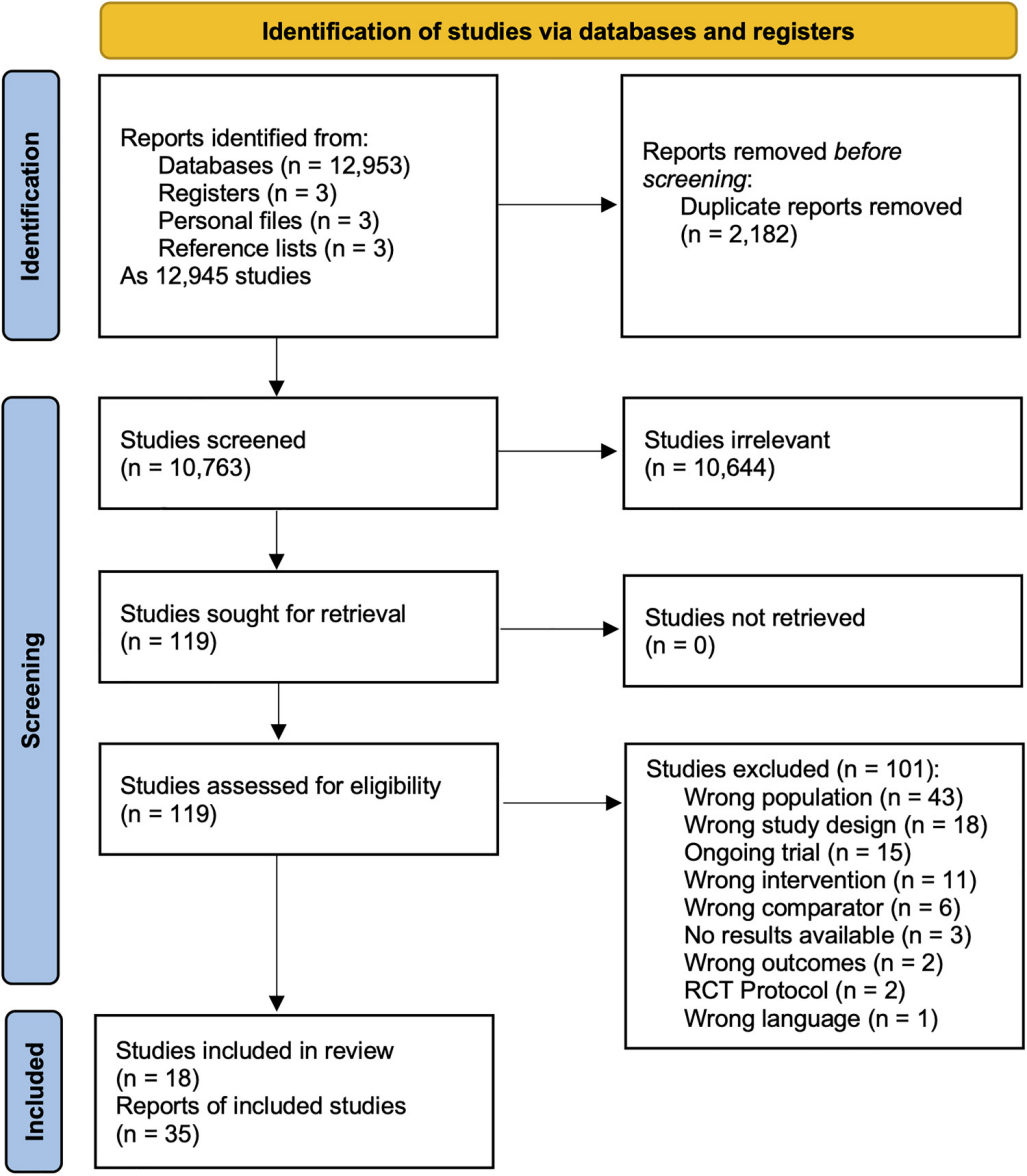
Preferred Reporting Items for Systematic reviews and Meta-Analyses flow diagram.

Our search identified 12 945 studies represented by 12 962 records (Figure 1). Of the 10 763 titles and abstracts screened, 119 studies were deemed appropriate to assess during the full text stage. Studies that initially appeared to meet eligibility criteria during full-text screening are summarized with the reason for exclusion in eTable 9. A total of 15 ongoing trials that may meet eligibility criteria upon their completion were identified as trial registry records (eTable 10). Eighteen studies met eligibility criteria and were included (eTable 11).

**Figure 2.**
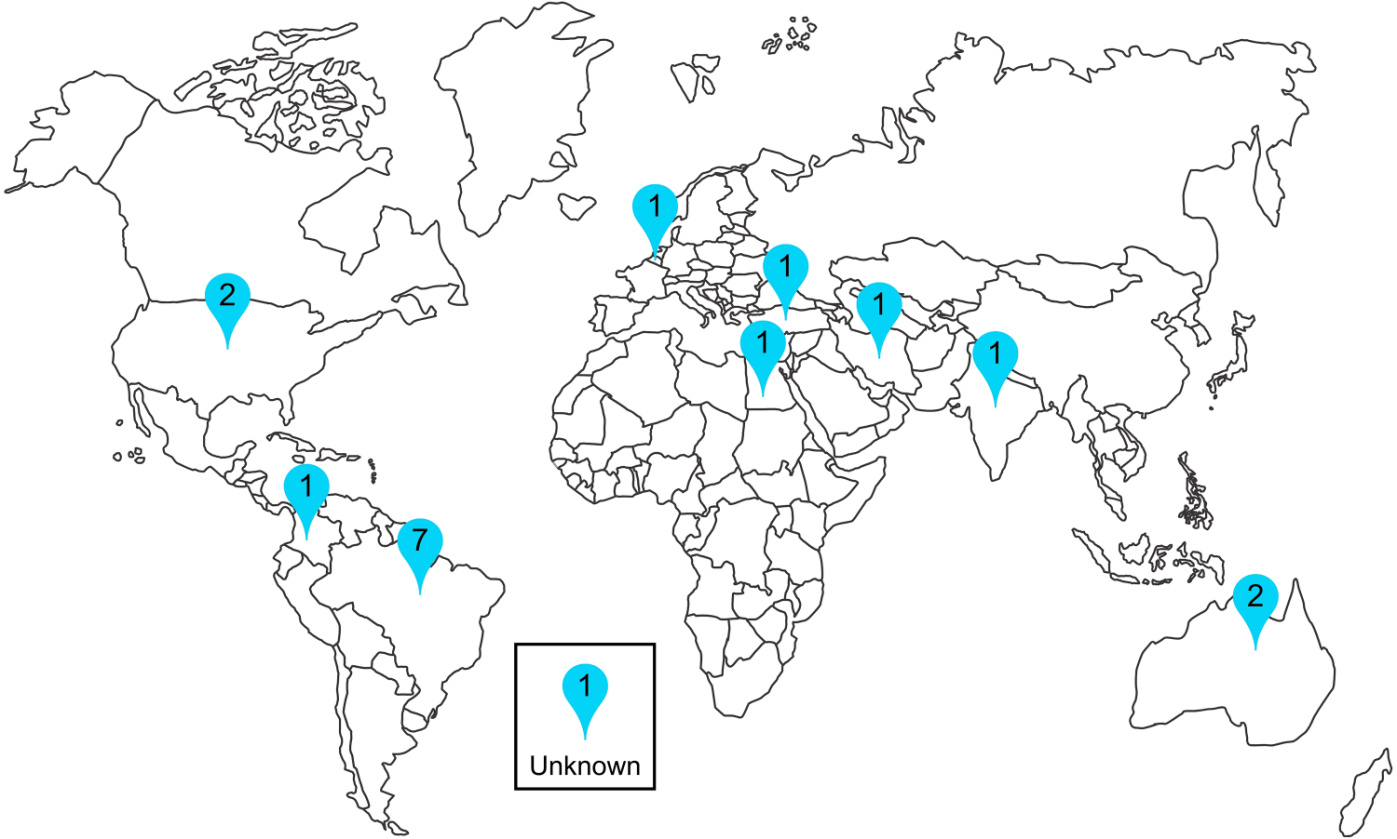
Map of the world with number of studies by country of origin.

Most studies originated from Brazil (Figure 2) and were single-centered (Table 1). Only 2 studies initiated IMT after weaning from IMV was completed; all other studies initiated IMT while participants still required mechanical ventilatory support.

**Table 1.** Characteristics of Included Studies.

Included studies, n studies	18
*Study details*	
Date of publication, n studies (%)	
2010-2014	9 (50)
2015-2019	3 (17)
2020-2024	6 (33)
Language, n studies (%)	
English	17 (94)
Portuguese	1 (6)
French	0 (0)
Number of centers, n studies (%)	
Single	17 (94)
Not reported	1 (6)
Timing of IMT initiation, n studies (%)	
During IMV	16 (89)
After weaning from IMV	2 (11)
Participants enrolled	
Total, N	901
Median (first, third quartiles)	43.5 (21, 70)
Participant baseline data reported	
Total, N^a^	831
Median (first, third quartiles)	36 (15, 70)
Outcomes assessed, n studies (%)	
Physical function	3 (17)
Mortality	8 (44)
Length of stay (hospital)	1 (6)
Length of stay (ICU)	4 (22)
IMV weaning time	9 (50)
Reintubation rate	6 (33)
Dyspnea	2 (11)
Maximal inspiratory pressure	18 (100)
Maximal expiratory pressure	2 (11)
Respiratory endurance	2 (11)

^a^ Two studies did not report baseline data for any participants; thus, results represent total participants from 16 studies.

### Physical Function

**Figure 3.**
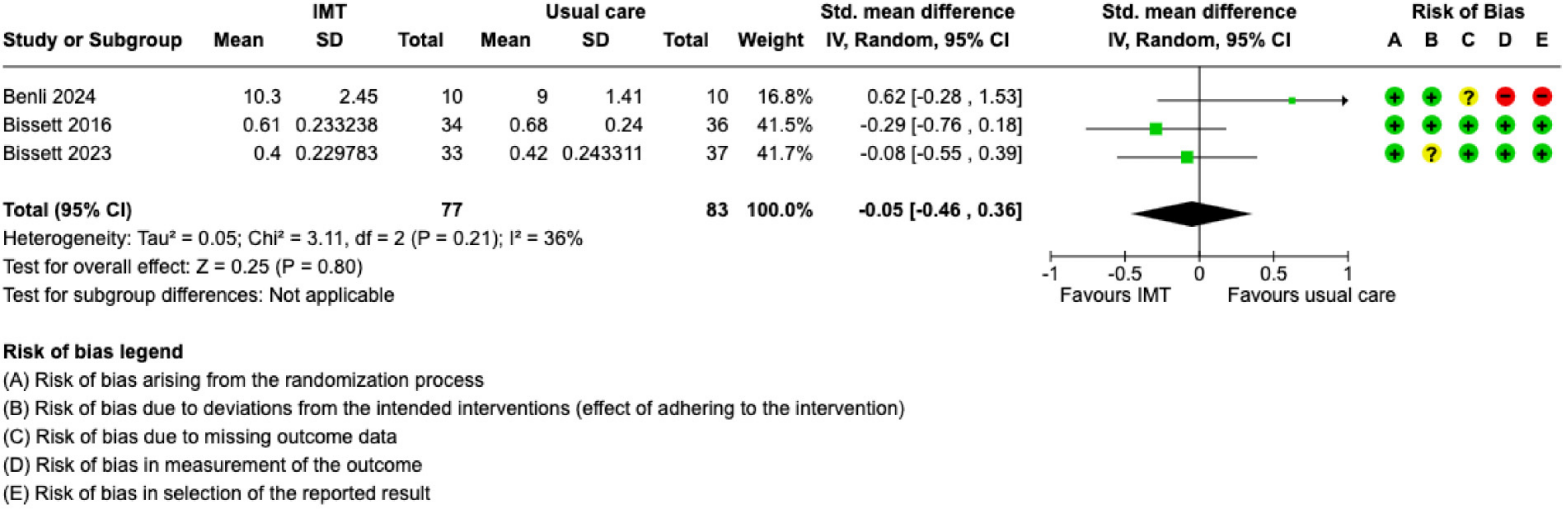
Forest plot of the standardized mean difference for physical function after completion of allocated treatment. IMT = inspiratory muscle training; SD = standard deviation; CI = confidence interval; IMV = invasive mechanical ventilation; + = low risk of bias; ? = some concerns for risk of bias; – = high risk of bias.

Three studies assessed and reported the effects of IMT on physical function. The Acute Care Index of Function^18^ was used in two trials^19,20^ and the Physical Function in Intensive Care Test^21^ was used in one trial.^22^ Physical function was assessed at enrollment and after completing the IMT protocol; no study assessed the outcome at ICU discharge, hospital discharge, or beyond. When combined, post-IMT treatment outcomes included 160 participants and demonstrated a SMD of −0.05 (95% CI: −0.46 to 0.36) (Figure 3). IMT may have no effect on physical function, however, the evidence is very uncertain (Table 2). Subgroup analyses of IMT initiation time (eFigure 1), IMV duration (eFigure 2), and treatment fidelity (eFigure 3) demonstrated no differences between subgroups. Since studies did not report results by age or diagnostic groups, we could not conduct physical function subgroup analyses for these characteristics.

**Table 2.** Summary of Findings Table.

Certainty assessment							№ of patients		Effect		Certainty	Importance
№ of studies	Study design	Risk of bias	Inconsistency	Indirectness	Imprecision	Other considerations	IMT	Usual care	Relative (95% CI)	Absolute (95% CI)		
Physical function												
3	randomised trials	serious^a^	serious^b^	not serious	very serious^c^	none	77	83	–	SMD **0.05 SD lower** (0.46 lower to 0.36 higher)	⨁◯◯◯ Very low^a,b,c^	CRITICAL
Respiratory endurance (assessed with: Fatigue Resistance Index)												
2	randomised trials	not serious	serious^b^	not serious	very serious^c^	none	67	73	–	MD **0** (0.18 lower to 0.19 higher)	⨁◯◯◯ Very low^b,c^	IMPORTANT
Dyspnea (assessed with: Modified Borg Scale)												
2	randomised trials	not serious	serious^d^	not serious	very serious^c^	none	67	73	–	**0.08 lower** (1.11 lower to 0.95 higher)	⨁◯◯◯ Very low^c,d^	IMPORTANT
ICU length of stay (assessed with: Days)												
4	randomised trials	very serious^e^	serious^d^	not serious	very serious^c^	none	83	87	–	**0.17 higher** (1.43 lower to 1.77 higher)	⨁◯◯◯ Very low^c,d,e^	IMPORTANT
Hospital length of stay (assessed with: Days)												
1	randomised trials	serious^f^	not serious	not serious	very serious^g^	none	10	10	–	MD **0.8 higher** (2.49 lower to 4.09 higher)	⨁◯◯◯ Very low^f,g^	IMPORTANT
Maximal inspiratory pressure (assessed with: centimeters of water)												
13	randomised trials	very serious^h^	not serious	not serious	not serious	publication bias strongly suspected^i^	348	368	–	**6.7 higher** (4.87 higher to 8.52 higher)	⨁◯◯◯ Very low^h,i^	NOT IMPORTANT
Ventilator weaning time (assessed with: Days)												
9	randomised trials	very serious^h^	serious^b^	not serious	not serious	none	248	258	–	MD **1.66 lower** (3 lower to 0.33 lower)	⨁◯◯◯ Very low^b,h^	IMPORTANT
Mortality												
8	randomised trials	very serious^j^	not serious	not serious	not serious	none	33/278 (11.9%)	54/303 (17.8%)	**RR 0.62** (0.42 to 0.91)	**68 fewer per 1000** (from 103 fewer to 16 fewer)	⨁⨁◯◯ Low^j^	CRITICAL
Reintubation												
6	randomised trials	very serious^k^	not serious	not serious	not serious	none	45/220 (20.5%)	62/228 (27.2%)	**RR 0.73** (0.54 to 0.99)	**73 fewer per 1000** (from 125 fewer to 3 fewer)	⨁⨁◯◯ Low^k^	IMPORTANT
Maximal expiratory pressure (assessed with: centimeters of water)												
1	randomised trials	serious^l^	not serious	not serious	very serious^g^	none	38	39	–	MD **3 higher** (2.15 lower to 8.15 higher)	⨁◯◯◯ Very low^g,l^	NOT IMPORTANT

IMT = inspiratory muscle training; CI = confidence interval; MD = mean difference; RR = risk ratio; SMD = standardised mean difference.

Explanations

^a^ High risk of bias in outcome measurement and selective reporting in 1 trial. Some concerns for risk of bias for deviations from intended intervention and missing outcome data in 1 trial each.

^b^ Clinical and statistical heterogeneity

^c^ <400 participants across included studies

^d^ Clinical heterogeneity

^e^ High risk of bias in 1 trial for deviations from the intended interventions. Several instances with some concern for risk of bias across 3 studies including all domains except outcome measurement.

^f^ Some concern for risk of bias related to missing outcome data and selective reporting.

^g^ 1 study with <400 participants

^h^ High risk of bias related to deviations from intended intervention in >5 trials. Some concern for risk of bias in multiple studies across all domains except outcome measurement.

^i^ Non-symmetrical funnel plot (see eFigure 28)

^j^ High risk of bias related to deviations from intended intervention in 1 trial. Some concerns for risk of bias in multiple studies across all domains except outcome measurement.

^k^ High risk of bias in 1 trial and some concerns in 1 trials related to concerns for deviations from intended intervention. Concerns for selective reporting in 4 trials. Concern for missing outcome data in 1 trial.

^l^ Some concerns for risk of bias related to missing outcome data and selective reporting.

### Maximal Inspiratory Pressure

All included studies assessed MIP.^19,20,22–37^ Results from five studies^27,29,32,33,35^ could not be included in our meta-analysis because mean, standard deviation and/or sample size were not reported; several unsuccessful attempts were made to contact study authors for clarification. Among these five studies, two reported higher MIP scores among intervention groups compared to control groups,^29,32^ and one reported no difference between groups.^33^ Additionally, two studies reported no within-group pre- and post-MIP differences.^27,35^

**Figure 4.**
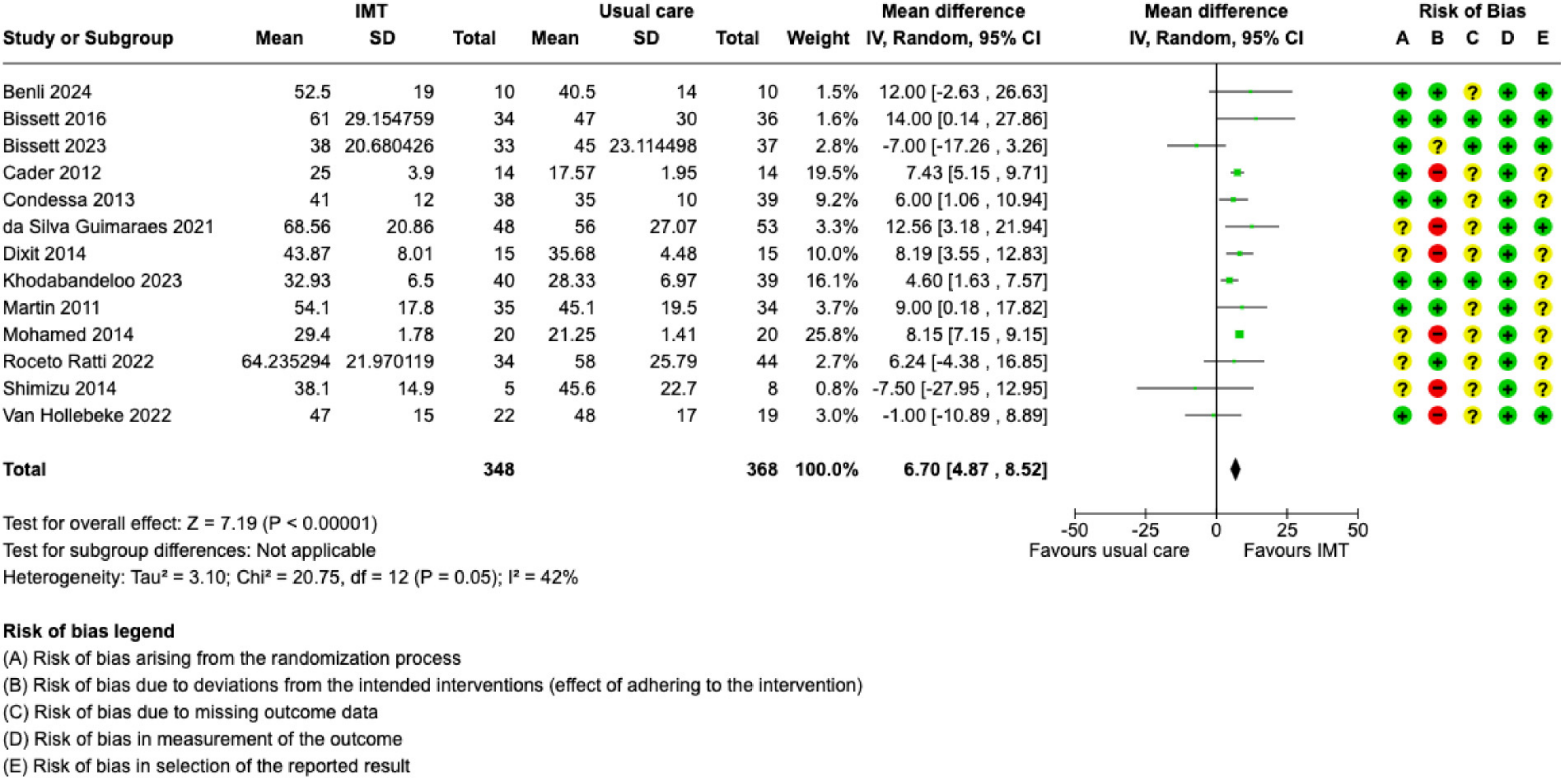
Forest plot of the maximal inspiratory pressure mean difference after completion of allocated treatment. IMT = inspiratory muscle training; SD = standard deviation; CI = confidence interval; IMV = invasive mechanical ventilation; + = low risk of bias; ? = some concerns for risk of bias; – = high risk of bias.

When we combined data from 716 participants, we found IMT may increase MIP (MD = 6.70 cmH_2_O, 95% CI: 4.87 to 8.52) (Figure 4), but the evidence is very uncertain (Table 2). Subgroup analysis of IMT initiation time (eFigure 4) found no difference between subgroups. Those with less than 7 days of IMV duration had higher MIP scores compared to those with 7 or more days of IMV (eFigure 5). IMT was associated with improved MIP in studies where treatment fidelity was at least 80%, but not in those where treatment fidelity was less than 80% (eFigure 6). Since studies did not report results by age or diagnostic groups, we could not conduct MIP subgroup analyses for these characteristics.

### Maximal Expiratory Pressure

Two studies evaluated MEP,^24,27^ but due to the format of reporting, we could only report between group differences from one study.^27^ As such, with data from only one study, we could not conduct meta- or subgroup analyses. Among 77 participants, IMT had no effect on MEP (MD = 3.00 cmH_2_O, 95% CI −2.15 to 8.15) (eFigure 7), but the certainty of evidence is very low (Table 2).

### Mortality

**Figure 5.**
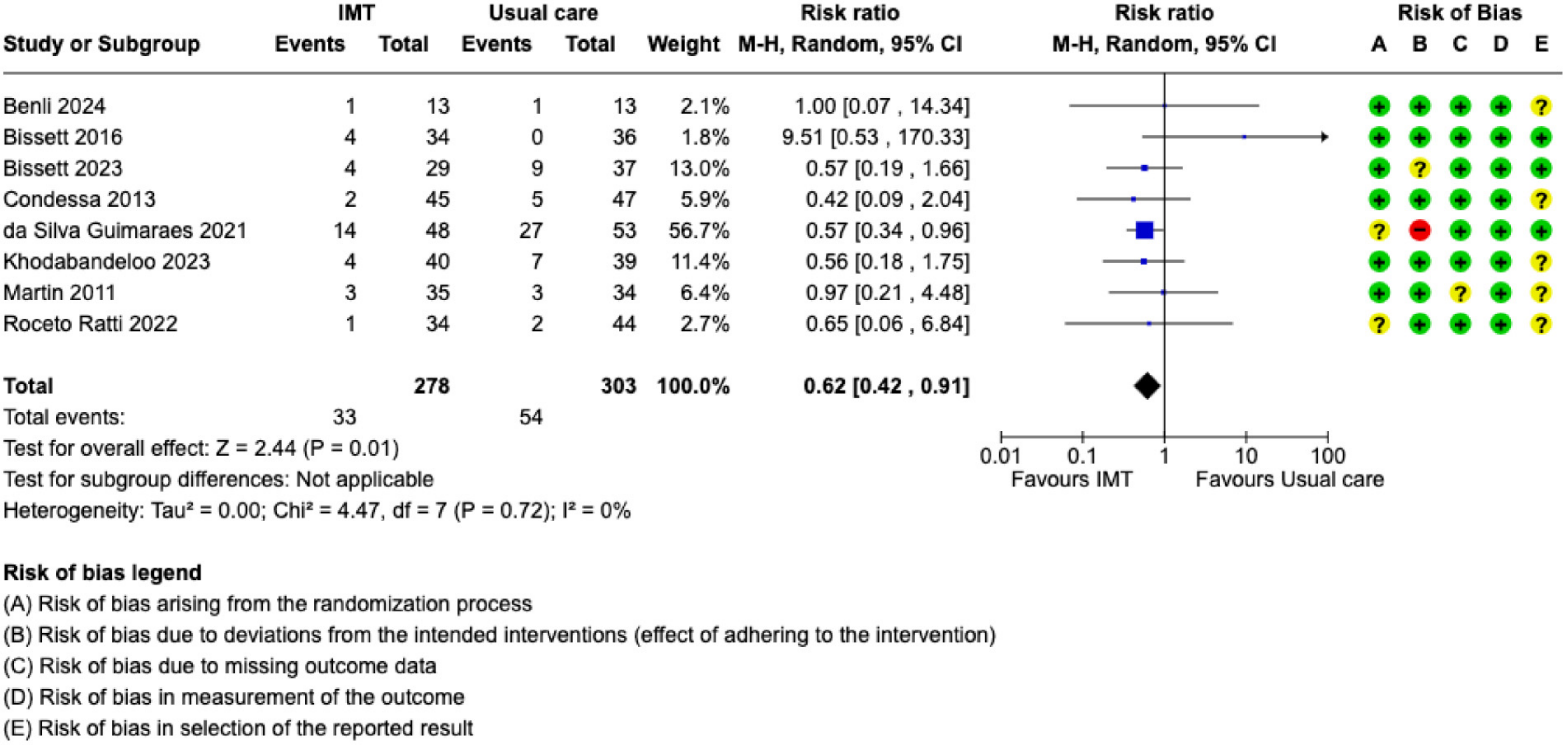
Forest plot of the risk ratio of mortality. IMT = inspiratory muscle training; SD = standard deviation; CI = confidence interval; IMV = invasive mechanical ventilation; M-H = Mantel-Haenszel; + = low risk of bias; ? = some concerns for risk of bias; – = high risk of bias.

Eight studies assessed and reported mortality.^19,20,22,24,25,28,31,37^ No study assessed mortality beyond hospital discharge. Due to inconsistent reporting, it was not possible to discern reports of hospital and ICU mortality, thus we considered all mortality reports as hospital mortality. Meta-analysis of results from 581 participants demonstrated that hospital mortality risk may be reduced among participants randomized to the IMT interventions (RR = 0.62, 95% CI: 0.42 to 0.91) (Figure 5); however, the certainty of evidence is low (Table 2). Subgroup analysis of IMT initiation time found that IMT initiated while the patient required IMV was associated with lower mortality compared to initiated after IMV was discontinued (eFigure 8). IMV duration (eFigure 9) and treatment fidelity (eFigure 10) subgroups demonstrated no differences. Since studies did not report results by age or diagnostic groups, we could not conduct mortality subgroup analyses for these characteristics.

### Length of Stay

#### ICU

Four studies reported ICU length of stay.^19,20,22,29^ One publication reported length of stay as median and first-third quartile values,^20^ which could not be combined in a meta-analysis. However, the study authors provided the mean and standard deviation values upon email communication. When combined, data from 170 participants demonstrated that IMT has no effect on ICU length of stay (MD = 0.17, 95% CI: −1.43 to 1.77) (eFigure 11), however, the evidence is very uncertain (Table 2). Subgroup analyses of IMT initiation time (eFigure 12), IMV duration (eFigure 13), and treatment fidelity (eFigure 14) demonstrated no differences between subgroups. Since studies did not report results by age or diagnostic groups, we could not conduct ICU length of stay subgroup analyses for these characteristics

#### Hospital

Hospital length of stay was only reported in one study^22^ which precluded meta- and subgroup analyses. Among 20 participants, IMT had no effect on hospital length of stay (MD = 0.80, 95% CI: −2.49 to 4.09) (eFigure 15), but the certainty of evidence is very low (Table 2).

### Invasive Mechanical Ventilator Weaning Time

Nine studies assessed IMV weaning time among a combined 506 participants.^23–26,28,29,31,33,34^ Meta-analysis demonstrated that IMT may reduce IMV weaning time (MD = −1.66 days, 95% CI: −3.00 to −0.33) (eFigure 16), however, the evidence is very uncertain (Table 2). Subgroup analysis (eFigure 17) demonstrated that weaning time decreased in those who received IMV for 7 or more days but not for those with less than 7 days of IMV. Subgroup analyses for IMT initiation time and treatment fidelity could not be conducted due to no studies being within either subgroup. Additionally, studies did not report IMV weaning time by age or diagnostic groups, so we could not conduct these subgroup analyses.

### Reintubation Rate

Six studies assessed reintubation rate.^19,20,24,28,33,35^ Due to narrative reporting and our inability to locate contact information for study authors, data from one study could not be included in our meta-analysis.^35^ Among 448 participants, IMT may reduce the risk of reintubation (RR = 0.73, 95% CI: 0.54 to 0.99) (eFigure 18), but the quality of evidence is low (Table 2). Additionally, Shrestha et al reported there was no between group difference in reintubation rate.^35^ Subgroup analyses found IMT initiation during IMV (eFigure 19), 7 or more days of IMV (eFigure 20), and less than 80% treatment fidelity (eFigure 21) were associated with a decreased risk of reintubation. Since studies did not report reintubation rate by age or diagnostic groups, we could not conduct these subgroup analyses.

### Dyspnea

Two studies^19,20^ assessed dyspnea in a combined 140 participants using the Modified Borg Dyspnea Scale.^38^ Dyspnea was assessed at enrollment and after completing the IMT protocol; no study assessed the outcome at ICU discharge, hospital discharge, or beyond. IMT may have no effect on post-intervention dyspnea (MD = −0.08, 95% CI: −1.11 to 0.95) (eFigure 22), but the evidence is very uncertain (Table 2). Subgroup analyses of IMT initiation time (eFigure 23) and treatment fidelity (eFigure 24) demonstrated no differences between subgroups. A subgroup analysis could not be completed by IMV duration because none of the two studies had a duration under 7 days. Additionally, subgroup analyses could not be completed by age or diagnosis because studies did not report results according to these variables.

### Respiratory Endurance

Two studies^19,20^ assessed respiratory endurance using the Fatigue Resistance Index^39^ after completing the IMT protocol. When data from the 140 participants were combined, IMT appeared to have no effect on post-intervention respiratory endurance (MD = 0.00, 95% CI −0.18 to 0.19) (eFigure 25), but the evidence is very uncertain (Table 2). Subgroup analyses of IMT initiation time (eFigure 26) and treatment fidelity (eFigure 27) demonstrated no differences between subgroups. A subgroup analysis could not be completed by IMV duration because neither of the studies had a duration under 7 days. Furthermore, subgroup analyses were not completed by age or diagnosis because studies did not report results according to these variables.

## Discussion

We comprehensively assessed the effects of IMT in adults admitted to the ICU who required at least 24 h of IMV. We found that IMT had no effect on physical function, ICU or hospital length of stay, dyspnea or respiratory endurance however, the certainty of evidence for these outcomes was either low or very low. Our findings suggest IMT may lead to higher MIP scores, reduced mortality, reduced IMV weaning time, and reduced reintubation rates; similarly, the certainty of these results was either low or very low.

Only 3 studies assessed the effects of IMT on physical function.^18–20^ In a 2017 modified-Delphi study, an international panel recommended physical function be included in a core outcome set for survivors of acute respiratory failure.^40,41^ A core outcome set is an agreed upon group of outcomes which should be measured and reported in all trials within a particular area of practice.^42^ Furthermore, physical function is regarded as a patient-important outcome^43^ which further supports its inclusion in trials of ICU physical rehabilitation. However, no studies assessed physical function after ICU discharge. Given the physical disability that can persist at 5 years after ICU discharge,^4^ long term follow-up is needed to understand the impact of IMT on functional status. Future studies need to consider the contribution of IMT beyond ICU and hospital discharge to maximize inspiratory muscle recovery. This could include the addition of home-based IMT as part of a post-hospital rehabilitation intervention. Home-based IMT has been shown to improve exercise capacity in similar populations.^44^

The reduction in diaphragmatic strength begins within hours of initiating IMV^1^ which indicates the need to begin targeted interventions early to prevent this decline. Over the past several decades, physical rehabilitation has emerged as an important component of ICU care to optimize physical functioning.^45^ However, identified barriers to physical rehabilitation in the ICU include the presence of an endotracheal tube and concerns for risk of line removal.^46^ IMT may be more amenable to delivery in situations where more active forms of physical rehabilitation (eg, transfer training) are contraindicated, such as in the presence of unstable lower extremity fractures and femoral sheaths.^47^ Given the positive impacts of IMT on physical function in people with post-COVID^48^ and post-stroke,^49^ IMT has the potential to prevent the physical decline associated with IMV as an adjunct to physical rehabilitation in the critically ill population. This review identified that IMT may have no effect on physical function, however this was based on 3 studies of low certainty. Future high quality RCTs are required to improve the precision of the effect estimate for IMT on physical function.

The eligibility criteria for most included studies required that patients were at least somewhat alert to facilitate participation in the IMT intervention. This is consistent with the physical rehabilitation literature, which has identified agitation, delirium and decreased level of alertness as a barrier to physical rehabilitation.^46^ Uniquely, Da Silva Guimarães et al^25^ had significantly more patients in the control group with a lower level of alertness compared to the intervention group. However, the authors conducted a post-hoc analysis and found that alertness did not have an impact on ventilator weaning success.^25^ As IMT requires active cooperation from the patient, its use in people with agitation and delirium may be challenging.

Our results suggest that IMT can effectively reduce mortality in patients who required at least 24 h of IMV. However, this strict interpretation should be cautioned. The IMT intervention was initiated in most studies either after the discontinuation of IMV or after patients were ready to initiate weaning from mechanical ventilation. As such, to meet eligibility criteria for the included studies, patients were likely already on a trajectory towards recovery, which may not have been altered even if IMT had not been initiated. Patients who were admitted to the ICU with more severe illness and thus a higher risk of death may not have met the eligibility criteria for these studies, which introduces a survivorship bias that is important to consider in the interpretation of the effects of IMT on mortality in this review.

To obtain true skeletal muscle hypertrophy, research suggests at least 18 resistive training sessions are required.^50^ Most studies reported their planned intervention or the treatment fidelity of the intervention such that it was not possible to determine the number of sessions that a patient underwent.^23–28,32–37^ Of those studies that reported their intervention plan and/or treatment fidelity such that the number of sessions a participant received could be interpreted,^19,20,22,29–31^ none reached the 18 session threshold. As such, the changes in MIP may not represent true respiratory muscle hypertrophy and, instead, may be caused by neural adaptation of the respiratory muscles^51,52^; however, further research is required. Additionally, sham-IMT has been shown to cause neural adaptation in healthy older adults,^53^ which may account for a potential training effect in the sham-IMT control groups.^35–37^ This could potentially reduce the likelihood of detecting a significant difference in outcomes between intervention and control groups.

## Strengths and Limitations

This review has limitations. We were unable to contact some authors to clarify outcome data to facilitate inclusion in meta-analyses. Due to small sample sizes among the included studies, only 901 participants were included within this review. Only studies reported in three languages were eligible for inclusion. Our review also has strengths. A priori, this review was registered with PROSPERO and its methods reported in a published protocol.^12^ We developed our search strategy in consultation with a health sciences librarian. We conducted the review with methods consistent with the Cochrane Handbook for Systematic Reviews and Intervention^11^ and reported it according to the PRISMA statement.^10^ This review was also led by experienced ICU physiotherapy clinicians with formal research training, which optimized the interpretation of included studies.

## Conclusion

We found that physical function was not affected by IMT, however, the results are very uncertain with limited sample sizes among included studies. Our review suggests that IMT may reduce reintubation risk and IMV weaning time. With the improved survivorship of individuals with critical illness, a focus of clinical research should include interventions to optimize patient-important outcomes, like physical function, after recovery. Future high quality RCTs are needed to improve the precision of the effect estimate of IMT on physical function.

## Supplemental Material

sj-docx-1-jic-10.1177_08850666251317473 - Supplemental material for The Effects of Inspiratory Muscle Training in Critically ill Adults: A Systematic Review and Meta-Analysis Supplemental material, sj-docx-1-jic-10.1177_08850666251317473 for The Effects of Inspiratory Muscle Training in Critically ill Adults: A Systematic Review and Meta-Analysis by Christopher Farley, Ana Oliveira, Dina Brooks and Anastasia N. L. Newman in Journal of Intensive Care Medicine
