# Supplementary material for: The Effects of Inspiratory Muscle Training in Critically ill Adults: A Systematic Review and Meta-Analysis
Source: J Intensive Care Med. 2025 Feb 9;41(4):263–73. doi: 10.1177/08850666251317473 (PMC13004845; doi:10.1177/08850666251317473)
Supplement: sj-docx-1-jic-10.1177_08850666251317473 - Supplemental material for The Effects of Inspiratory Muscle Training in Critically ill Adults: A Systematic Review and Meta-Analysis [file sj-docx-1-jic-10.1177_08850666251317473.docx]

**The effects of inspiratory muscle training in critically ill adults: A systematic review and meta-analysis**

**Supplemental file**

Christopher Farley, PT, MSc^a^; Ana Oliveira, PT, PhD^a,b^; Dina Brooks, PT, PhD^a,c,d,e,f^; Anastasia N.L. Newman, PT, PhD^a^

^a^ School of Rehabilitation Science, Faculty of Health Science, McMaster University, Hamilton ON, Canada

^b^ Lab 3R – Respiratory Research and Rehabilitation Laboratory, School of Health Sciences (ESSUA) and Institute of Biomedicine

^c^ Department of Respiratory Medicine, West Park Healthcare Centre, Toronto ON, Canada

^d^ Department of Physical Therapy, Faculty of Medicine, University of Toronto, Toronto ON, Canada

^e^ Rehabilitation Sciences Institute, School of Graduate Studies, University of Toronto, Toronto ON, Canada

^f^ Department of Medicine, Faculty of Medicine, University of Toronto, Toronto ON, Canada

**Corresponding author**: Dr. Anastasia N. L. Newman, [newmanan@mcmaster.ca](mailto:newmanan@mcmaster.ca), 1400 Main Street West Institute for Applied Health Sciences (IAHS) Building - Room 429, Hamilton, ON L8S 1C7

**eTable 1**. Preferred Reporting Items for Systematic reviews and Meta-Analyses (PRISMA) 2020 statement checklist

| **Section and Topic** | **Item #** | **Checklist item** | **Location where item is reported** |
| --- | --- | --- | --- |
| **TITLE** | | |  |
| **Title** | **1** | **Identify the report as a systematic review.** | Title |
| **ABSTRACT** | | |  |
| **Abstract** | **2** | **See the PRISMA 2020 for Abstracts checklist.** | Abstract |
| **INTRODUCTION** | | |  |
| **Rationale** | **3** | **Describe the rationale for the review in the context of existing knowledge.** | Introduction (paragraph 1-2) |
| **Objectives** | **4** | **Provide an explicit statement of the objective(s) or question(s) the review addresses.** | Introduction (paragraph 3) |
| **METHODS** | | |  |
| **Eligibility criteria** | **5** | **Specify the inclusion and exclusion criteria for the review and how studies were grouped for the syntheses.** | Eligibility criteria (paragraph 1) |
| **Information sources** | **6** | **Specify all databases, registers, websites, organisations, reference lists and other sources searched or consulted to identify studies. Specify the date when each source was last searched or consulted.** | Information sources (paragraph 1) |
| **Search strategy** | **7** | **Present the full search strategies for all databases, registers and websites, including any filters and limits used.** | Search strategy (paragraph 1); eTables 2-8 |
| **Selection process** | **8** | **Specify the methods used to decide whether a study met the inclusion criteria of the review, including how many reviewers screened each record and each report retrieved, whether they worked independently, and if applicable, details of automation tools used in the process.** | Selection process (paragraph 1) |
| **Data collection process** | **9** | **Specify the methods used to collect data from reports, including how many reviewers collected data from each report, whether they worked independently, any processes for obtaining or confirming data from study investigators, and if applicable, details of automation tools used in the process.** | Data collection (paragraph 1) |
| **Data items** | **10a** | **List and define all outcomes for which data were sought. Specify whether all results that were compatible with each outcome domain in each study were sought (e.g. for all measures, time points, analyses), and if not, the methods used to decide which results to collect.** | Data items (paragraph 1) |
|  | **10b** | **List and define all other variables for which data were sought (e.g. participant and intervention characteristics, funding sources). Describe any assumptions made about any missing or unclear information.** | Data items (paragraph 1) |
| **Study risk of bias assessment** | **11** | **Specify the methods used to assess risk of bias in the included studies, including details of the tool(s) used, how many reviewers assessed each study and whether they worked independently, and if applicable, details of automation tools used in the process.** | Risk of bias assessment (paragraph 1) |
| **Effect measures** | **12** | **Specify for each outcome the effect measure(s) (e.g. risk ratio, mean difference) used in the synthesis or presentation of results.** | Data analysis (paragraph 2) |
| **Synthesis methods** | **13a** | **Describe the processes used to decide which studies were eligible for each synthesis (e.g. tabulating the study intervention characteristics and comparing against the planned groups for each synthesis (item #5)).** | Data analysis |
|  | **13b** | **Describe any methods required to prepare the data for presentation or synthesis, such as handling of missing summary statistics, or data conversions.** | Data collection process (paragraph 1); Data analysis (paragraph 4) |
|  | **13c** | **Describe any methods used to tabulate or visually display results of individual studies and syntheses.** | Data analysis |
|  | **13d** | **Describe any methods used to synthesize results and provide a rationale for the choice(s). If meta-analysis was performed, describe the model(s), method(s) to identify the presence and extent of statistical heterogeneity, and software package(s) used.** | Data analysis (paragraph 3) |
|  | **13e** | **Describe any methods used to explore possible causes of heterogeneity among study results (e.g. subgroup analysis, meta-regression).** | Data analysis (paragraph 5) |
|  | **13f** | **Describe any sensitivity analyses conducted to assess robustness of the synthesized results.** | Not applicable |
| **Reporting bias assessment** | **14** | **Describe any methods used to assess risk of bias due to missing results in a synthesis (arising from reporting biases).** | Data analysis (paragraph 6) |
| **Certainty assessment** | **15** | **Describe any methods used to assess certainty (or confidence) in the body of evidence for an outcome.** | Certainty assessment (paragraph 1) |
| **RESULTS** | | |  |
| **Study selection** | **16a** | **Describe the results of the search and selection process, from the number of records identified in the search to the number of studies included in the review, ideally using a flow diagram.** | Results (paragraph 1); Figure 1 |
|  | **16b** | **Cite studies that might appear to meet the inclusion criteria, but which were excluded, and explain why they were excluded.** | eTable 9 |
| **Study characteristics** | **17** | **Cite each included study and present its characteristics.** | eTable 11 |
| **Risk of bias in studies** | **18** | **Present assessments of risk of bias for each included study.** | Figure 3-5; eFigure 1-27; |
| **Results of individual studies** | **19** | **For all outcomes, present, for each study: (a) summary statistics for each group (where appropriate) and (b) an effect estimate and its precision (e.g. confidence/credible interval), ideally using structured tables or plots.** | Figure 3-5; eFigure 1-27; |
| **Results of syntheses** | **20a** | **For each synthesis, briefly summarise the characteristics and risk of bias among contributing studies.** | Table 2; Figure 3-5; eFigure 1-27 |
|  | **20b** | **Present results of all statistical syntheses conducted. If meta-analysis was done, present for each the summary estimate and its precision (e.g. confidence/credible interval) and measures of statistical heterogeneity. If comparing groups, describe the direction of the effect.** | Table 2; Figure 3-5; eFigure 1-27 |
|  | **20c** | **Present results of all investigations of possible causes of heterogeneity among study results.** | Table 2 |
|  | **20d** | **Present results of all sensitivity analyses conducted to assess the robustness of the synthesized results.** | Not applicable |
| **Reporting biases** | **21** | **Present assessments of risk of bias due to missing results (arising from reporting biases) for each synthesis assessed.** | Table 2; eFigure 28 |
| **Certainty of evidence** | **22** | **Present assessments of certainty (or confidence) in the body of evidence for each outcome assessed.** | Table 2 |
| **DISCUSSION** | | |  |
| **Discussion** | **23a** | **Provide a general interpretation of the results in the context of other evidence.** | Discussion (paragraph 2, 3, 6) |
|  | **23b** | **Discuss any limitations of the evidence included in the review.** | Strengths and limitations (paragraph 1) |
|  | **23c** | **Discuss any limitations of the review processes used.** | Strengths and limitations (paragraph 1) |
|  | **23d** | **Discuss implications of the results for practice, policy, and future research.** | Discussion (paragraph 2, 3) |
| **OTHER INFORMATION** | | |  |
| **Registration and protocol** | **24a** | **Provide registration information for the review, including register name and registration number, or state that the review was not registered.** | Methods (paragraph 1) |
|  | **24b** | **Indicate where the review protocol can be accessed, or state that a protocol was not prepared.** | Methods (paragraph 1) |
|  | **24c** | **Describe and explain any amendments to information provided at registration or in the protocol.** | Data analysis (paragraph 5) |
| **Support** | **25** | **Describe sources of financial or non-financial support for the review, and the role of the funders or sponsors in the review.** | Funding |
| **Competing interests** | **26** | **Declare any competing interests of review authors.** | None |
| **Availability of data, code and other materials** | **27** | **Report which of the following are publicly available and where they can be found: template data collection forms; data extracted from included studies; data used for all analyses; analytic code; any other materials used in the review.** | To be published on Open Science Framework at study completion |

***From:*  Page MJ, McKenzie JE, Bossuyt PM, Boutron I, Hoffmann TC, Mulrow CD, et al. The PRISMA 2020 statement: an updated guideline for reporting systematic reviews. BMJ 2021;372:n71. doi: 10.1136/bmj.n71**

**eTable 2**. Medline (OVID interface, 1946 - April 13, 2024) search strategy

| **Concept** | **Search terms** |
| --- | --- |
| Randomized controlled trial[^1^](#_ENREF_1) | 1. randomized controlled trial.pt.  2. controlled clinical trial.pt.  3. randomized.ab.  4. placebo.ab.  5. drug therapy.fs.  6. randomly.ab.  7. trial.ab.  8. groups.ab.  9. 1 or 2 or 3 or 4 or 5 or 6 or 7 or 8 |
| Critical illness | 10. exp animals/ not humans.sh.  11. 9 not 10  12. intensive care units/ or burn units/ or coronary care units/ or respiratory care units/  13. (intensive care or burn unit* or coronary care unit* or respiratory care unit* or ICU or ICUs).mp.  14. Critical Illness/  15. critical* ill*.mp.  16. Critical Care/  17. critical care.mp.  18. airway management/ or airway extubation/ or intubation, intratracheal/ or respiration, artificial/ or ventilator weaning/ or tracheostomy/  19. (airway management or extubat* or intubat* or ventilator* or mechanical* ventilat* or tracheostomy or artificial respiration).mp.  20. Respiratory Insufficiency/  21. (respirat* insufficiency or respirat* failure).mp.  22. 12 or 13 or 14 or 15 or 16 or 17 or 18 or 19 or 20 or 21 |
| Inspiratory muscle training | 23. exercise therapy/ or endurance training/ or resistance training/  24. (exercise therapy or endurance training or resistance training).mp.  25. (muscle* training adj2 (inspiratory or respiratory)).mp.  26. (muscle* strength* adj2 (inspiratory or respiratory)).mp.  27. physical therapy modalities/ or exercise movement techniques/ or breathing exercises/  28. (physical therap* or physiotherap* or exercise movement or breathing exercise*).mp.  29. 23 or 24 or 25 or 26 or 27 or 28 |
| Combined concepts and limits | 30. 11 and 22 and 29  31. limit 30 to humans |

**eTable 3**. Embase (OVID interface, 1974 – April 13, 2024) search strategy

| **Concept** | **Search terms** |
| --- | --- |
| Randomized controlled trial[^2^](#_ENREF_2) | 1. randomized controlled trial/  2. controlled clinical study/  3. or/1-2  4. random$.ti,ab.  5. randomization/  6. intermethod comparison/  7. placebo.ti,ab.  8. (compare or compared or comparison).ti.  9. ((evaluated or evaluate or evaluating or assessed or assess) and (compare or compared or comparing or comparison)).ab.  10. (open adj label).ti,ab.  11. ((double or single or doubly or singly) adj (blind or blinded or blindly)).ti,ab.  12. double blind procedure/  13. parallel group$1.ti,ab.  14. (crossover or cross over).ti,ab.  15. ((assign$ or match or matched or allocation) adj5 (alternate or group$1 or intervention$1 or patient$1 or subject$1 or participant$1)).ti,ab.  16. (assigned or allocated).ti,ab.  17. (controlled adj7 (study or design or trial)).ti,ab.  18. (volunteer or volunteers).ti,ab.  19. human experiment/  20. trial.ti.  21. or/4-20  22. 21 not 3  23. (random$ adj sampl$ adj7 (cross section$ or questionnaire$1 or survey or database$1)).ti,ab. not (comparative study/ or controlled study/ or randomi?ed controlled.ti,ab. or randomly assigned.ti,ab.)  24. Cross-sectional study/ not (randomized controlled trial/ or controlled clinical study/ or controlled study/ or randomi?ed controlled.ti,ab. or control group$1.ti,ab.)  25. (((case adj control$) and random$) not randomi?ed controlled).ti,ab.  26. (Systematic review not (trial or study)).ti.  27. (nonrandom$ not random$).ti,ab.  28. Random field$.ti,ab.  29. (random cluster adj3 sampl$).ti,ab.  30. (review.ab. and review.pt.) not trial.ti.  31. we searched.ab. and (review.ti. or review.pt.)  32. update review.ab.  33. (databases adj4 searched).ab.  34. (rat or rats or mouse or mice or swine or porcine or murine or sheep or lambs or pigs or piglets or rabbit or rabbits or cat or cats or dog or dogs or cattle or bovine or monkey or monkeys or trout or marmoset$1).ti. and animal experiment/  35. Animal experiment/ not (human experiment/ or human/)  36. or/23-35  37. 22 not 36 |
| Critical illness | 38. intensive care unit/ or exp burn unit/ or exp coronary care unit/ or exp medical intensive care unit/ or exp neurological intensive care unit/ or exp surgical intensive care unit/  39. intensive care/  40. (intensive care or critical care or burn unit* or coronary care unit* or respiratory care unit* or ICU or ICUs).mp.  41. exp critical illness/  42. critical* ill*.mp.  43. artificial ventilation/ or invasive ventilation/ or positive pressure ventilation/ or pressure controlled ventilation/ or pressure support ventilation/ or volume controlled ventilation/  44. ((artificial or invasive or positive pressure or pressure controlled or pressure support or volume controlled or mechanical*) adj ventilat*).mp.  45. intubation/ or respiratory tract intubation/  46. extubation/ or tracheal extubation/  47. tracheostomy/  48. (intubat* or extubat* or tracheostom*).mp.  49. respiratory failure/  50. (respirat* insufficiency or respirat* failure).mp.  51. 38 or 39 or 40 or 41 or 42 or 43 or 44 or 45 or 46 or 47 or 48 or 49 or 50 |
| Inspiratory muscle training | 52. exercise/ or breathing exercise/ or endurance training/ or resistance training/ or muscle training/  53. (exercis* or (training adj (endurance or resistance or breathing or muscl*))).mp.  54. physiotherapy/  55. (physiotherap* or physical therap*).mp.  56. 52 or 53 or 54 or 55 |
| Combined concepts and limits | 57. 37 and 51 and 56  58. limit 57 to human |

**eTable 4**. Emcare (OVID interface, 1995 – April 13, 2024) search strategy

| **Concept** | **Search terms** |
| --- | --- |
| Randomized controlled trial | 1. randomization/  2. randomized controlled trial/ or controlled clinical trial/  3. clinical trial/  4. trial.mp.  5. groups.mp.  6. random*.mp.  7. 1 or 2 or 3 or 4 or 5 or 6 |
| Critical illness | 8. intensive care unit/ or exp burn unit/ or exp coronary care unit/ or exp medical intensive care unit/ or exp neurological intensive care unit/ or exp surgical intensive care unit/  9. intensive care/  10. (intensive care or critical care or burn unit* or coronary care unit* or respiratory care unit* or ICU or ICUs).mp.  11. critical illness/  12. critical* ill*.mp.  13. artificial ventilation/ or invasive ventilation/ or positive pressure ventilation/ or pressure controlled ventilation/ or pressure support ventilation/ or volume controlled ventilation/  14. ((artificial or invasive or positive pressure or pressure controlled or pressure support or volume controlled or mechanical*) adj ventilat*).mp.  15. intubation/ or respiratory tract intubation/  16. extubation/ or tracheal extubation/  17. tracheostomy/  18. (intubat* or extubat* or tracheostom*).mp.  19. respiratory failure/  20. (respirat* insufficiency or respirat* failure).mp.  21. 8 or 9 or 10 or 11 or 12 or 13 or 14 or 15 or 16 or 17 or 18 or 19 or 20 |
| Inspiratory muscle training | 22. exercise/ or breathing exercise/ or endurance training/ or muscle exercise/ or resistance training/  23. (exercis* or (training adj (endurance or resistance or breathing or muscl*))).mp.  24. physiotherapy/  25. (physiotherap* or physical therap*).mp.  26. 22 or 23 or 24 or 25 |
| Combined concepts and limits | 27. 7 and 21 and 26  28. limit 27 to human |

**eTable 5**. AMED (OVID interface, 1985 – April 13, 2024) search strategy

| **Concept** | **Search terms** |
| --- | --- |
| Randomized controlled trial | 1. clinical trials/ or randomized controlled trials/ or random allocation/  2. controlled clinical trial.mp.  3. random*.mp.  4. trial.mp.  5. groups.mp.  6. 1 or 2 or 3 or 4 or 5 |
| Critical illness | 7. critical care/ or intensive care/  8. (intensive care or critical care or ICU or ICUs).mp.  9. critical illness/  10. critical* ill*.mp.  11. Ventilators mechanical/ or Respiration artificial/  12. ((artificial or invasive or positive pressure or pressure controlled or pressure support or volume controlled or mechanical*) adj ventilat*).mp.  13. intubation/  14. (intubat* or extubat* or tracheostom*).mp.  15. Respiratory Insufficiency/  16. (respirat* insufficiency or respirat* failure).mp.  17. 7 or 8 or 9 or 10 or 11 or 12 or 13 or 14 or 15 or 16 |
| Inspiratory muscle training | 18. breathing exercises/ or chest physiotherapy/ or exercise therapy/  19. (exercise therapy or endurance training or resistance training).mp.  20. (muscle* training adj2 (inspiratory or respiratory)).mp.  21. (muscle* strength* adj2 (inspiratory or respiratory)).mp.  22. physiotherapists/  23. (physical therap* or physiotherap*).mp.  24. 18 or 19 or 20 or 21 or 22 or 23 |
| Combined concepts and limits | 25. 6 and 17 and 24 |

**eTable 6**. CINAHL (EBSCOhost interface, 1981 – April 13, 2024) search strategy

| **Concept** | **Search terms** |
| --- | --- |
| Randomized controlled trial[^3^](#_ENREF_3) | S23 S22 NOT S21  S22 S1 OR S2 OR S3 OR S4 OR S5 OR S6 OR S7 OR S8 OR S9 OR S10 OR S11 OR S12 OR S13 OR S14 OR S15  S21 S19 NOT S20  S20 MH (human)  S19 S16 OR S17 OR S18  S18 TI (animal model*)  S17 MH (animal studies)  S16 MH animals+  S15 AB (cluster W3 RCT)  S14 MH (crossover design) OR MH (comparative studies)  S13 AB (control W5 group)  S12 PT (randomized controlled trial)  S11 MH (placebos)  S10 MH (sample size) AND AB (assigned OR allocated OR control)  S9 TI (trial)  S8 AB (random*)  S7 TI (randomized OR randomized)  S6 (MH "Cluster Sample")  S5 (MH "Pretest-Posttest Design")  S4 (MH "Random Assignment")  S3 (MH "Single-Blind Studies")  S2 (MH "Double-Blind Studies")  S1 (MH "Randomized Controlled Trials") |
| Critical illness | S44 S24 OR S25 OR S26 OR S27 OR S28 OR S29 OR S30 OR S31 OR S32 OR S33 OR S34 OR S35 OR S36 OR S37 OR S38 OR S39 OR S40 OR S41 OR S42 OR S43  S43 "respirat* insufficiency" or "respirat* failure"  S42 (MH "Respiratory Failure")  S41 "intubat*"  S40 "ventilat*"  S39 "artificial* respirat*"  S38 (MH "Intubation") OR (MH "Intubation, Intratracheal")  S37 (MH "Ventilator Patients")  S36 (MH "Respiration, Artificial") OR (MH "Positive Pressure Ventilation") OR (MH "Ventilator Weaning")  S35 (MH "Critically Ill Patients")  S34 "critical* ill*"  S33 (MH "Critical Illness")  S32 "critical care"  S31 (MH "Critical Care")  S30 "ICUs"  S29 ""ICU""  S28 "burn unit*"  S27 "respiratory care unit*"  S26 "coronary care unit*"  S25 "intensive care"  S24 (MH "Intensive Care Units") OR (MH "Coronary Care Units") OR (MH "Respiratory Care Units") OR (MH "Burn Units") |
| Inspiratory muscle training | S54 S45 OR S46 OR S47 OR S48 OR S49 OR S50 OR S51 OR S52 OR S53  S53 "respiratory muscle train*"  S52 "inspiratory muscle train*"  S51 (MH "Chest Physical Therapy")  S50 "physiotherap*"  S49 "physical therap*"  S48 "resistance train*"  S47 "breathing exercis*"  S46 ""muscle strength*""  S45 (MH "Muscle Strengthening") OR (MH "Breathing Exercises") OR (MH "Resistance Training") OR (MH "Physical Therapy") OR (MH "Therapeutic Exercise") |
| Combined concepts and limits | S55 S23 AND S44 AND S54 – (Limiters HUMAN) |

**eTable 7**. CENTRAL (Cochrane library interface, inception – April 13, 2024) search strategy

| **Concept** | **Search terms** |
| --- | --- |
| Randomized controlled trial | #1 MeSH descriptor: [Random Allocation] this term only  #2 MeSH descriptor: [Randomized Controlled Trial] explode all trees  #3 random*  #4 groups  #5 trial  #6 #1 or #2 or #3 or #4 or #5 |
| Critical illness | #7 MeSH descriptor: [Intensive Care Units] this term only  #8 MeSH descriptor: [Critical Care] this term only  #9 MeSH descriptor: [Critical Illness] explode all trees  #10 MeSH descriptor: [Positive-Pressure Respiration] this term only  #11 MeSH descriptor: [Ventilators, Mechanical] this term only  #12 MeSH descriptor: [Respiratory Insufficiency] this term only  #13 Intensive care unit*  #14 Critical care  #15 Critical* ill*  #16 Ventilat*  #17 Artificial respirat*  #18 Respirat* failure  #19 Respirat* insufficiency  #20 ICU  #21 #7 or #8 or #9 or #10 or #11 or #12 or #13 or #14 or #15 or #16 or #17 or #18 or #19 or #20 |
| Inspiratory muscle training | #22 MeSH descriptor: [Breathing Exercises] this term only  #23 MeSH descriptor: [Muscle Strength] this term only  #24 MeSH descriptor: [Exercise Therapy] this term only  #25 MeSH descriptor: [Physical Therapy Modalities] this term only  #26 MeSH descriptor: [Resistance Training] this term only  #27 Inspiratory muscle train*  #28 Respiratory muscle train*  #29 Chest physiotherap*  #30 Chest physical therap*  #31 Resistance train*  #32 Breathing exercis*  #33 Muscle strength*  #34 #22 or #23 or #24 or #25 or #26 or #27 or #28 or #29 or #30 or #31 or #32 or #33 |
| Combined concepts and limits | #35 #6 AND #21 AND #34 |

**eTable 8**. ClinicalTrials.gov search strategy

| **Text field** | **Search terms** |
| --- | --- |
| Condition or disease field | (critical* ill*) OR (critical care) OR (intensive care) OR (ICU*) OR (ventilat*) OR (respirat* failure) OR (respirat* insufficiency) or (ICU) |
| Intervention/treatment field | (IMT) or (inspiratory muscle train*) OR (respiratory muscle train*) OR (chest physiotherap*) or (chest physical therap*) or (resistance train*) or (breathing exercis*) or (muscle strength*) |

**eTable 9.** Summary of excluded studies which appear to meet eligibility criteria

| **Author Year** | **Reason for exclusion** | | | | | **Notes** |
| --- | --- | --- | --- | --- | --- | --- |
|  | **Wrong population** | **Wrong intervention** | **Wrong comparator** | **Wrong outcome** | **Other** |  |
| Aldrich 1989[^4^](#_ENREF_4) |  |  |  |  | X | Not a RCT |
| Bento 2023[^5^](#_ENREF_5) | X |  |  |  |  | Participants did not require invasive mechanical ventilation for at least 24 hours |
| Caruso 2005[^6^](#_ENREF_6) |  | X |  |  |  | Did not utilize a threshold device for inspiratory muscle training |
| Chang 2022[^7^](#_ENREF_7) | X |  |  |  |  | Admitted to a post-ICU care location |
| Dellweg 2017[^8^](#_ENREF_8) | X |  |  |  |  | Admitted to a post-ICU care location |
| Elbouhy 2014[^9^](#_ENREF_9) |  |  |  |  | X | Not randomized |
| Ferreira 2019[^10^](#_ENREF_10) |  |  | X |  |  | Comparator was not usual care |
| Koch 2020[^11^](#_ENREF_11) | X |  |  |  | X | Not critically ill, cross-over RCT |
| Reginault 2023[^12^](#_ENREF_12) |  |  | X |  |  | Comparator was not usual care |
| Smith 2014 |  |  |  |  | X | Cohort study |

*Legend:* RCT = randomized controlled trial; ICU = intensive care unit.

**eTable 10.** Details of ongoing trials

| **Registry** | **Registration number** | **Title** |
| --- | --- | --- |
| Brazilian Clinical Trial Registry | RBR-2z7mtr | Inspiratory muscle training in patients with traumatic brain injury undergoing prolonged mechanical ventilation: randomized controlled trial study |
| Brazilian Clinical Trial Registry | RBR-32vkkr | Inspiratory muscle training in intensive care unit patients |
| Chinese Clinical Trial Registry | ChiCTR2300071173 | The effect of inspiratory muscle training in difficult to wean patients with neuromuscular disease |
| Chinese Clinical Trial Registry | ChiCTR2300073637 | Based on best evidence perioperative respiratory muscle exercise program in patients undergoing cardiac surgery |
| ClinicalTrials.gov | NCT03758573 | Effectiveness inspirational muscle training (IMTversusMV) |
| ClinicalTrials.gov | NCT03908658 | Inspiratory muscle training and nasal high flow in difficult weaning |
| ClinicalTrials.gov | NCT04507451 | Respiratory muscle training in ICU patients |
| ClinicalTrials.gov | NCT04658498 | Improving our understanding of respiratory muscle training to facilitate weaning from mechanical ventilation in the ICU (TrainToWean) |
| ClinicalTrials.gov | NCT05271019 | Efficacy of early inspiratory muscle training in lung transplanted patients |
| ClinicalTrials.gov | NCT06046690 | Comparison of the effects of inspiratory muscle training methods in mechanically ventilated patients |
| ClinicalTrials.gov | NCT06167239 | Ventilator trigger sensitivity adjustment versus threshold inspiratory muscle training on arterial blood gases |
| ClinicalTrials.gov | NCT06210763 | IMT outcomes in sufferers of severe walking disabilities and intensive care unit acquired weakness |
| Iranian Registry of Clinical Trials | IRCT20230214057418N1 | The effect of respiratory muscle exercises on pulmonary functional indices and successful extubation in patients hospitalized in the intensive care unit |
| Pan African Clinical Trial Registry | PACTR201909644273809 | Effects of a threshold trainer on inspiratory muscle strength in mechanically ventilated patients: a randomized controlled trial |
| Pan African Clinical Trial Registry | PACTR202209768392751 | Ventilator inspiratory trigger sensitivity adjustment versus threshold device training on difficult to wean Guillain Barre patients |

**eTable 11.** Details of included studies

| **Author (year), country, funding** | **ICU type, hospital type, number of sites** | **Eligibility** | **Groups** | **N randomized, N with baseline characteristics reported** | **Age (years), Males (%)** | **Type of IMT device (model)** | **Treatment description** | | **Outcomes assessed (tool, if applicable)** |
| --- | --- | --- | --- | --- | --- | --- | --- | --- | --- |
|  |  |  |  |  |  |  | **Components** | **Fidelity** |  |
| **IMT initiated after IMV discontinued** |  |  |  |  |  |  |  |  |  |
| Benli (2024)[^13^](#_ENREF_13)  Turkey  None | Respiratory ICU  University hospital  1 site | 18-80 years; IMV ≥ 2 days; Sedation Agitation Score = 4; Hemodynamic stability | IMT + conventional PT | 13  10 | 64.10 (8.21)  8 (80) | Threshold device (PowerBreathe Medic Plus) | IMT at 30% of MIP for twice daily for 5 days. 30 breaths, 4 sets of 6-8 breaths | 95% of planned IMT and conventional PT sessions were completed | Physical function (PFIT)  MIP  ICU LOS  Hospital LOS  Mortality  Reintubation rate |
|  |  |  | Conventional PT | 13  10 | 62.8 (16.37)  8 (80) |  | Breathing exercises, thoracic expansion exercises, coughing and gradual mobilization, once daily for 5 days after extubation | 80% of planned conventional PT sessions were completed |  |
| Bissett (2016)[^14-16^](#_ENREF_14)  Australia  Canberra Hospital Private Practice Fund (2010) and Canberra Hospital Auxiliary Research Fund | NR  Tertiary hospital  1 site | ≥16 years; IMV ≥ 7 days; Successfully weaned from IMV (>48 hours); <7 days since successful weaning; RSAS score=4 | IMT + usual PT | 34  34 | 59 (16)  24 (71) | Threshold device (Respironics HS730) | IMT at 50% of MIP with rapid increase to highest tolerable intensity, for once daily for 2 weeks. 5 sets of 6 breaths | 85% of participants completed all planned IMT sessions  67% of participants completed >90% of planned IMT sessions  6% of participants completed ≤20% planned IMT sessions | Physical function (ACIF)  MIP  Dyspnea (mBorg)  Respiratory endurance (FSI)  ICU LOS  Mortality  Reintubation rate |
|  |  |  | Usual PT | 36  36 | 59 (13)  21 (58) |  | Assisted mobilization, secretion clearance, positive expiratory pressure techniques, deep breathing, and upper and lower limb exercises | No participants inadvertently received IMT |  |
| **IMT initiated during IMV** |  |  |  |  |  |  |  |  |  |
| Bissett (2023)[^17^](#_ENREF_17)  Australia  Canberra Hospital Private Practice Fund and Canberra Hospital Auxiliary Research Fund | Medical, surgical, and trauma ICU  Tertiary hospital  1 site | ≥16 years; IMV ≥ 7 days; RSAS score=4 | IMT + usual care | 33  33 | 60 (17)  18 (55) | Threshold device (Respironics HS730) | IMT at 50% of MIP with rapid increase to highest tolerable intensity, once daily (weekdays only) from randomization until 1 week following successful liberation from IMV, 5 sets of 6 breaths | 58% of participants completed >70% of planned IMT sessions  6% of participants completed ≤30% planned IMT sessions  Participants completed median 8 (Range: 2-67) sessions  71% of planned IMT sessions completed among all participants | Physical function (ACIF)  IMV weaning time  Dyspnea (mBorg)  MIP  Respiratory endurance (FSI)  ICU LOS  Mortality  Reintubation rate |
|  |  |  | Usual care | 37  37 | 59 (15)  23 (62) |  | Secretion clearance (percussion, hyperinflation, suction) | NR |  |
| Cader (2012)[^18-20^](#_ENREF_18)  Brazil  NR | NR  NR  1 site | IMV ≥ 48 hours in controlled mode; has a MIP of 20 cmH_2_O | IMT + conventional PT | 14  14 | 82 (4)  6 (43) | Threshold device (Philips Respironics) | IMT at 30% of MIP, increased by 10% daily, for 5 minutes, twice daily from start of IMV weaning to extubation | NR | IMV weaning time  MIP |
|  |  |  | Conventional PT | 14  14 | 81 (6)  7 (50) |  | Passive to active-assisted mobilization of limbs, chest compression, decompression traction chest, aspiration of the endotracheal tube and positioning | NR |  |
| Condessa (2013)[^21^](#_ENREF_21)  Brazil  Research and Event Inventive Fund | General ICU  University hospital  1 site | ≥18 years;  IMV ≥ 48 hours in controlled mode and ready for IMV weaning; hemodynamic stability | IMT + usual PT | 45  45 | 64 (17)  23 (51) | Threshold device (Respironics Threshold IMT) | IMT at 40% of MIP, twice daily from start of pressure-support ventilation, 5 sets of 10 breaths | Participants underwent IMT daily throughout weaning period. | IMV weaning time  MIP  MEP  Mortality |
|  |  |  | Usual PT | 47  47 | 65 (15)  28 (60) |  | Passive to active-assisted mobilization of limbs, chest compression with quick release at end-expiration, aspiration of the endotracheal tube and positioning | NR |  |
| Da Silva Guimarães (2021)[^22-25^](#_ENREF_22)  Brazil  Partially funded by the Universidade Estácio de Sá | General ICU  Military hospital  1 site | ≥3 failed weaning attempts or ≥7 days of weaning; cough reflex; medical stability | IMT + early mobilization and t-piece trials | 55  48 | 63 (16)  24 (50) | Tapered flow resistance device (POWER breathe K-5) | IMT using an electronic device, once daily, 5 days per week until weaned from IMV, 2 sets of 30 breaths | NR | MIP  Mortality |
|  |  |  | Early mobilization and t-piece trials | 55  53 | 69 (16)  25 (47) |  | Early mobilization and progressively lengthening t-piece trials, daily, until weaned from IMV | NR |  |
| Dixit (2014)[^26^](#_ENREF_26)  India  Self-funded | General ICU  University hospital  1 site | >18 years; SIMV, CPAP, SIMV+PS and PSV ventilator modes; conscious and cooperative; hemodynamic stability; MIP < 35 cmH_2_O | IMT + conventional PT | 15  NR | NR  NR | Threshold device (NR) | IMT at 30% of MIP, increased by 10% per day then adjusted to a RPE of 6-8/10, twice daily, 5 sets of 6 breaths until extubation | NR | IMV weaning time  MIP |
|  |  |  | Conventional PT | 15  NR | NR  NR |  | Positioning, diaphragmatic retraining and recruitment strategies, segmental and thoracic expansion exercises, percussions and vibrations, postural drainage, coughing, huffing, active and passive ROM | NR |  |
| Dos Santos Pascotini (2014)[^27^](#_ENREF_27)  Brazil  NR | NR  NR  1 site | ≥40 years; tracheostomy; undergoing IMV weaning | IMT + PT | 7  7 | 67.0 (13.9)  0 (0) | Threshold device (Respironics Threshold IMT) | IMT at 20% of MIP, once daily, 3 sets of 10 breaths until extubation | NR | MIP  MEP |
|  |  |  | PT | 7  7 | 72.4 (11.9)  3 (43) |  | Airway clearance techniques, aspiration, mobilization (i.e., shoulder, elbow, thigh and knee flexion and extension, shoulder and thigh abduction and adduction, ankles plantar flexion and lower limb stretching), 3 times daily | NR |  |
| Khodabandeloo (2023)[^28^](#_ENREF_28)^,^[^29^](#_ENREF_29)  Iran  None | NR  Teaching hospital  1 site | IMV for 1 week; RASS -1, 0 or +1; medical stability | IMT + conventional PT | 40  40 | 64.6 (15.3)  24 (60) | Threshold device (NR) | IMT at 50% of MIP, increased by 10% of MIP per day, once daily, 5 sets of 6 breaths | 87.5% of participants completed all planned IMT sessions | IMV weaning time  MIP  Mortality  Reintubation rate |
|  |  |  | Conventional PT | 39  39 | 67.4 (13.8)  17 (44) |  | Range of passive to active movements of limbs, vibrations, percussions, repositioning, for an average of 15 minutes, once daily | NR |  |
| Martin (2011)[^30-32^](#_ENREF_30)  United States of America  Funding provided by the National Institutes of Health. | General, medical, surgical, and burns ICU  University hospital  1 site | ≥18 years; following verbal commands; medically stable; assist control, SIMV or PS IMV modes; life expectancy of ≥12 months | IMT + breathing trials | 35  35 | 65.6 (11.7)  16 (46) | Threshold device (Respironics Threshold IMT) | IMT for 4 sets of 6 to 10 breaths, once daily, 5 days per week + breathing trials of reduced or no MV support | Mean (SD) of 16% (21) of study days on which participants unable to participate in IMT | MIP  Mortality |
|  |  |  | Sham IMT + breathing trials | 34  34 | 65.1 (10.7)  15 (44) |  | Sham IMT for 4 sets of 6 to 10 breaths, once daily, 5 days per week + breathing trials of reduced or no MV support | Mean (SD) of 15% (18) of study days on which participants unable to participate in Sham-IMT |  |
| Melo (2018)[^33^](#_ENREF_33)^,^[^34^](#_ENREF_34)  Brazil  NR | NR  NR  ~~1 site~~ | >18 years;  ~~IMV >7 days~~ ; non-collaborative neurocritical patients | ~~IMT + usual care~~ | ~~6~~  ~~NR~~ | NR  NR | ~~Tapered flow resistance device (POWER breathe K-5)~~ | ~~IMT at 50% of MIP, daily for 14 days~~ | NR | ~~IMV weaning time~~  ~~MIP~~  ~~ICU LOS~~ |
|  |  |  | ~~Usual care~~ | ~~4~~  ~~NR~~ | NR  NR |  | ~~Early mobilization and respiratory therapy for 14 days~~ | NR |  |
| Mohamed (2014)[^35^](#_ENREF_35)^,^[^36^](#_ENREF_36)  Egypt  NR | NR  University hospital  1 site | IMV ≥ 48 hours; medically stable; conscious and responding to commands; tolerate spontaneous breathing trials | IMT + usual chest PT | 20  20 | 54.8 (7.6)  11 (55) | NR (NR) | IMT at 30% of MIP, for 5 to 6 sets of 6 breaths, for total of 18-30 breaths, twice daily for about 12 sessions per week | NR | MIP |
|  |  |  | Usual chest PT | 20  20 | 55.9 (3.07)  10 (50) |  | 3 times daily, including manual hyperinflation, percussion, vibrations, suction, breathing exercises, muscle training of upper and lower limbs. | NR |  |
| Roceto Ratti (2022)[^37-39^](#_ENREF_37)  Brazil  NR | Internal medicine, trauma, surgical and neurology ICU  University hospital  1 site | ≥18 years; IMV ≥ 48 hours; tracheostomy; readiness for weaning | Automatic IMT + standard respiratory PT | 25  25 | 52 (17.3)  15 (59) | Tapered flow resistance device (POWERbreathe KH2) | IMT automatically adjusted according to the maximal effort during first 2 breaths of session, 3 sets of 10 repetitions, twice daily, 7 days per week | Median of 7.5 (4-12.5) IMT sessions | IMV weaning time  MIP  Mortality |
|  |  |  | Manual IMT + standard respiratory PT | 26  26 | 57 (15.57)  18 (70) |  | IMT at 30% of MIP, increased by10% daily, 3 sets of 10 repetitions, twice daily, 7 days per week | Median of 3.4 (2-8) IMT sessions |  |
|  |  |  | standard respiratory PT | 53  53 | 56 (18.29)  43 (81) |  | Bronchial hygiene, tracheal and oral cavity suction, physical training including bedside sitting, active and active-assisted exercises | NR |  |
| Saad (2014)[^40^](#_ENREF_40)  NR  NR | NR  NR  NR | IMV; tracheostomy | IMT + respiratory PT | 8  8 | NR  7 (87.5) | Tapered flow resistance device (POWERbreathe KH2) | IMT at 30% of initial MIP, increased by 10% daily, for 3 sets of 10 breaths | NR | MIP |
|  |  |  | T-tube + Respiratory PT | 11  11 | NR  8 (73) |  | T-tube until 48 hours of continuous nebulization | NR |  |
| Sandoval Moreno (2019)[^41^](#_ENREF_41)^,^[^42^](#_ENREF_42)  Colombia  Vice-Rectorate for Research  of Universidad del Valle | Level 4 ICU  NR  1 site | ≥18 years; IMV ≥ 48 hours; RASS of -1 or 0; medical stability | IMT + usual care | 62  62 | 61 (40-70)  33 (53) | Threshold device (Respironics Threshold IMT) | IMT at 50% of MIP, twice daily for 3 sets of 6-10 breaths, | NR | IMV weaning time  MIP  Reintubation rate |
|  |  |  | Usual care | 64  64 | 62 (47-72)  38 (59) |  | Respiratory physiotherapy, physical therapy and mechanical ventilation management | NR |  |
| Shimizu (2014)[^43^](#_ENREF_43)  Brazil  NR | NR  University hospital  1 site | >18 years; IMV ≥ 48 hours | IMT + T-tube | 5  5 | 65.40 (10.88)  NR | Threshold device (NR) | IMT at 50% of MIP, twice daily for 3 sets of 10 breaths, | NR | IMV weaning time  MIP |
|  |  |  | T-tube | 8  8 | 62.37 (14.41)  NR |  | Spontaneous breathing trials, 8 times daily, beginning at 5 minutes | NR |  |
| Shrestha (2014)[^44^](#_ENREF_44)  United States of America  RO1 NR011186 | NR  NR  1 site | Acute respiratory failure with IMV; failed two-hour spontaneous breathing trial, | IMT | 4  4 | 49 (16.9)  NR | Threshold device (Respironics Threshold IMT) | IMT, twice daily, for 5 minutes | 3 of 4 patients tolerated IMT well.  1 or 4 patients needed to return to ventilator due to dyspnea | MIP  Reintubation rate |
|  |  |  | Sham IMT | 3  3 | 54.33 (3.78)  NR |  | Sham device, twice daily, for 5 minutes | 100% of sham sessions were tolerated |  |
| Van Hollebeke (2022)[^45-47^](#_ENREF_45)  Belgium  Research Foundation Flanders (grant number G053721N) Research Foundation Flanders (FWO 12U5618N). | Medical and surgical ICU  NR  1 site | Failed IMV wean; able to follow verbal commands | IMT | 24  22 | 52 (18)  13 (59) | Tapered flow resistance device (POWERbreathe KH2) | IMT at 30% of MIP, increased to highest tolerable load, 4 sets of 6-8 repetitions | Completed 74% of planned sessions | MIP |
|  |  |  | Sham IMT | 23  19 | 64 (7)  9 (47) |  | Sham IMT at 10% of MIP, 4 sets of 6-8 repetitions | Completed 75% of planned sessions |  |

*Legend:* All values reported as mean (SD) or median (1^st^-3^rd^ quartiles). ICU = intensive care unit; IMT = inspiratory muscle training; IMV = invasive mechanical ventilation; PT = physical therapy; MIP = maximal inspiratory pressure; PFIT = Physical Function in Intensive Care Test; LOS = length of stay; NR = not reported; RSAS = Riker Sedation-Agitation Scale; ACIF = Acute Care Index of Function; mBorg = Modified Borg Dyspnea Scale; FSI = Fatigue Resistance Index; cmH_2_O = centimetres of water; MEP = maximal expiratory pressure; SIMV = synchronized intermittent mandatory ventilation; CPAP = continuous positive airway pressure; PS = pressure support; PSV = pressure support ventilation; RPE = rating of perceived exertion; ROM = range of motion; RASS = Richmond Agitation-Sedation Scale; SD = standard deviation.


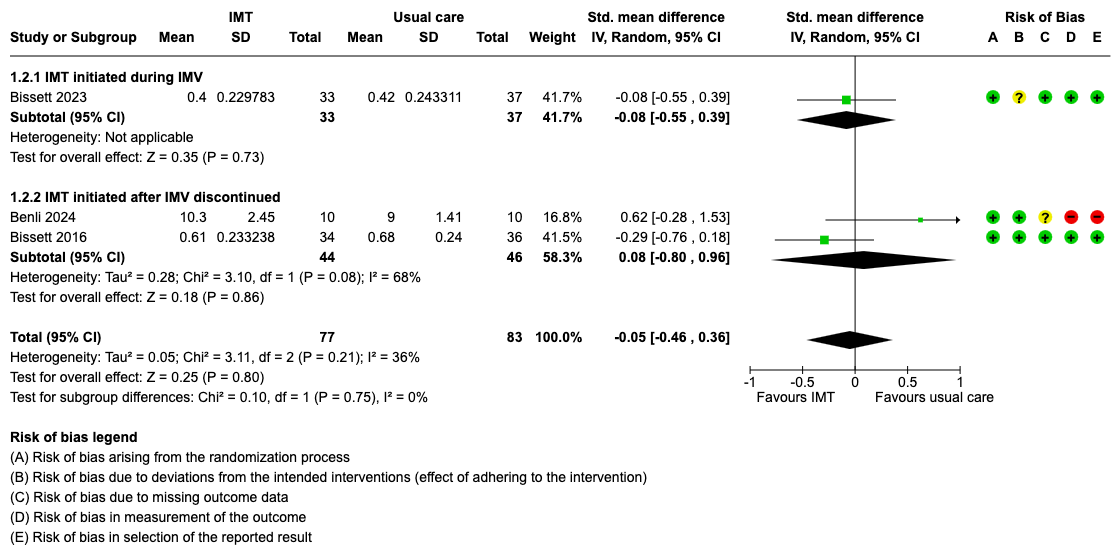


**eFigure 1**. Forest plot of the standardized mean difference for physical function after completion of allocated treatment by IMT initiation time. IMT = inspiratory muscle training; SD = standard deviation; CI = confidence interval; IMV = invasive mechanical ventilation; + = low risk of bias; ? = some concerns for risk of bias; – = high risk of bias.

**
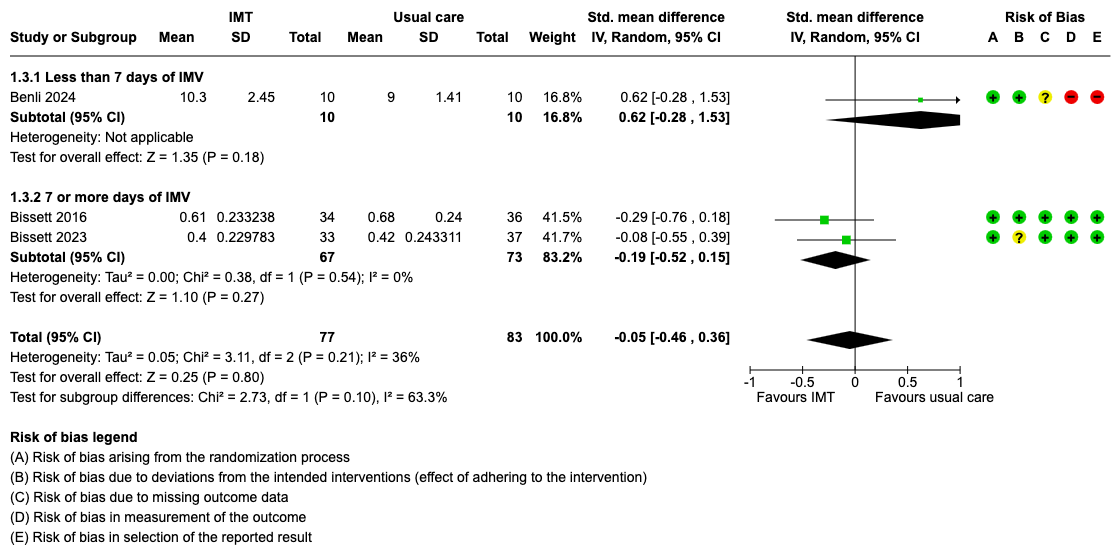
**

**eFigure 2**. Forest plot of the standardized mean difference for physical function after completion of allocated treatment by invasive mechanical ventilation duration. IMT = inspiratory muscle training; SD = standard deviation; CI = confidence interval; IMV = invasive mechanical ventilation; + = low risk of bias; ? = some concerns for risk of bias; – = high risk of bias.


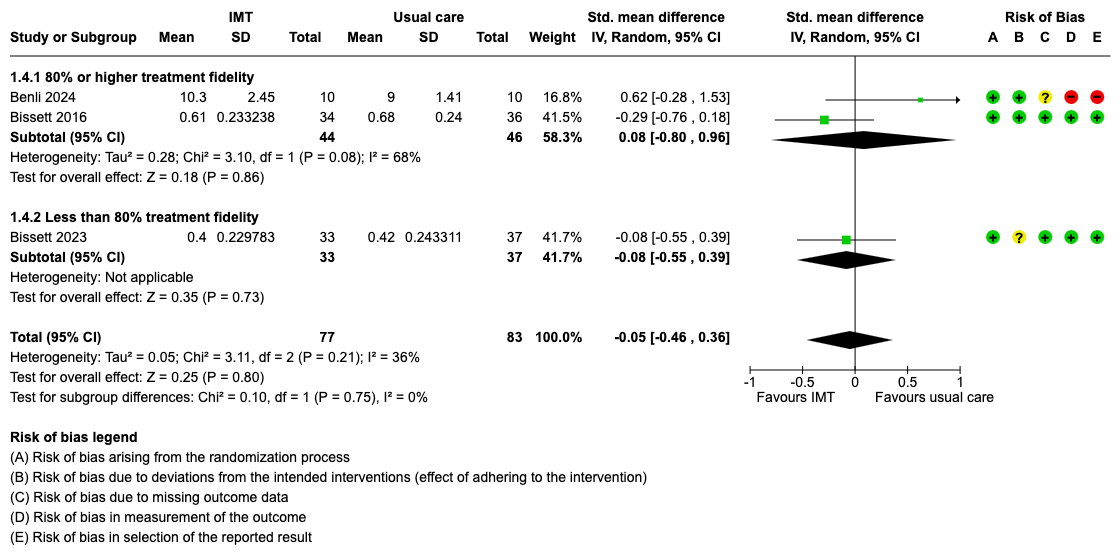


**eFigure 3**. Forest plot of the standardized mean difference for physical function after completion of allocated treatment by treatment fidelity. IMT = inspiratory muscle training; SD = standard deviation; CI = confidence interval; IMV = invasive mechanical ventilation; + = low risk of bias; ? = some concerns for risk of bias; – = high risk of bias.

**
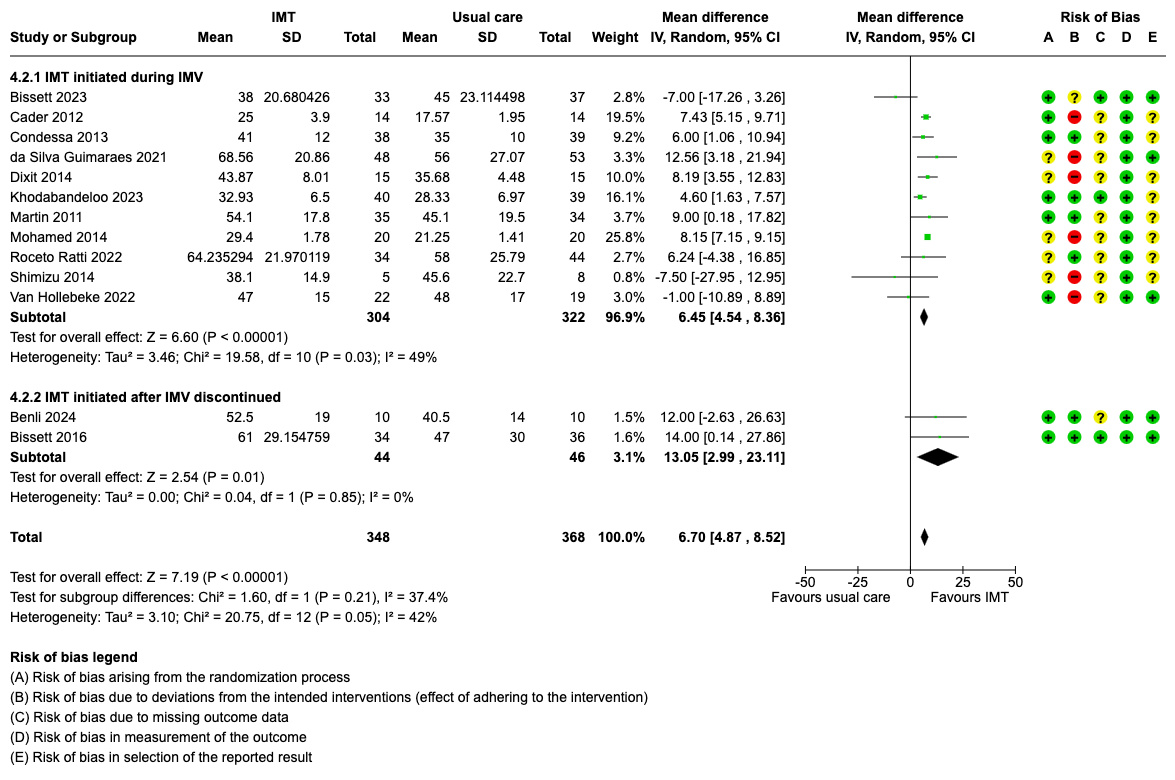
**

**eFigure 4**. Forest plot of the maximal inspiratory pressure mean difference after completion of allocated treatment by IMT initiation time. IMT = inspiratory muscle training; SD = standard deviation; CI = confidence interval; IMV = invasive mechanical ventilation; + = low risk of bias; ? = some concerns for risk of bias; – = high risk of bias.

**
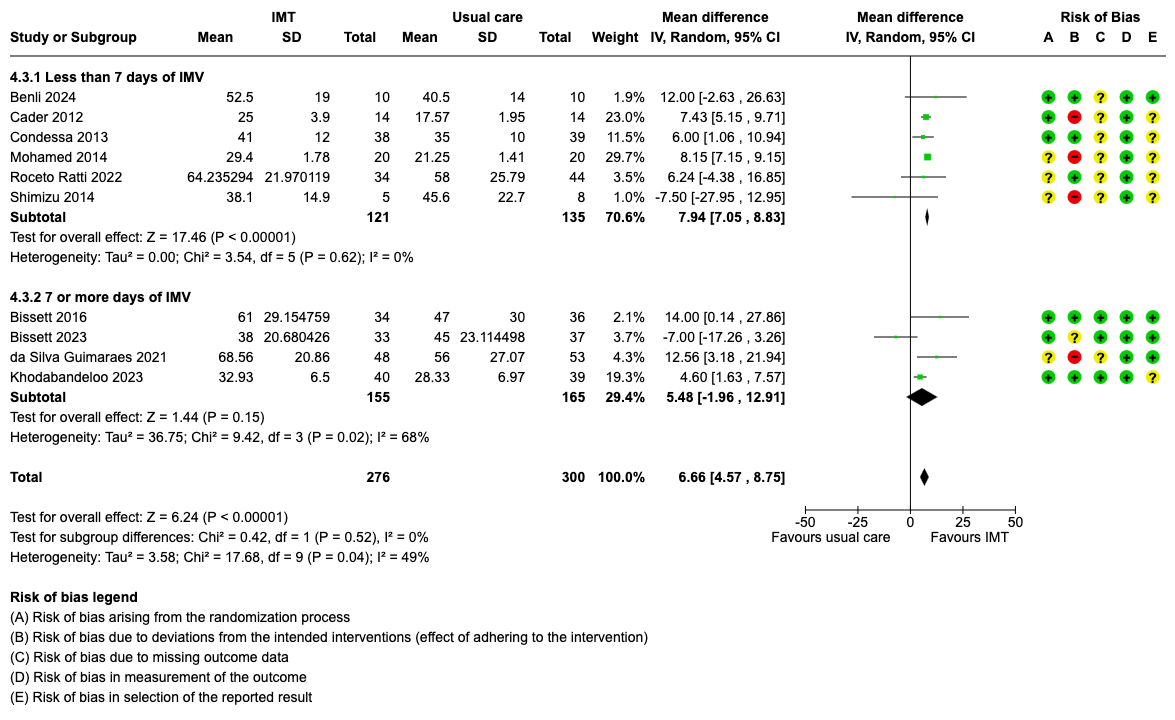
**

**eFigure 5**. Forest plot of the maximal inspiratory pressure mean difference after completion of allocated treatment by invasive mechanical ventilation duration. IMT = inspiratory muscle training; SD = standard deviation; CI = confidence interval; IMV = invasive mechanical ventilation; + = low risk of bias; ? = some concerns for risk of bias; – = high risk of bias.

**
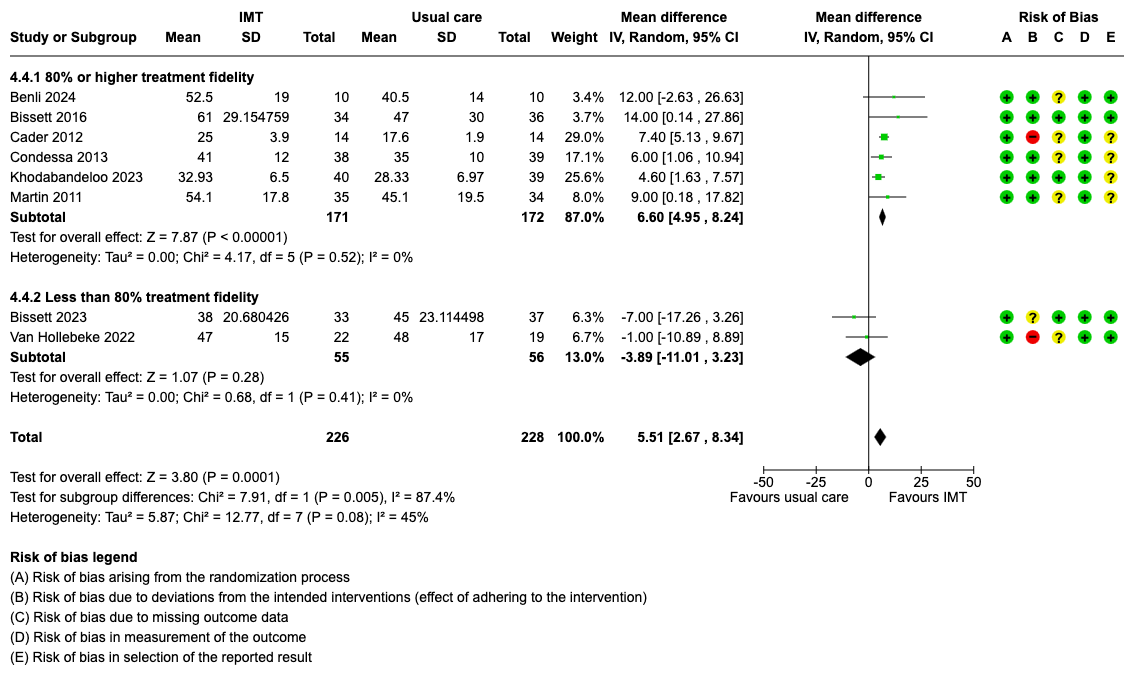
**

**eFigure 6**. Forest plot of the maximal inspiratory pressure mean difference after completion of allocated treatment by treatment fidelity. IMT = inspiratory muscle training; SD = standard deviation; CI = confidence interval; IMV = invasive mechanical ventilation; + = low risk of bias; ? = some concerns for risk of bias; – = high risk of bias.

**
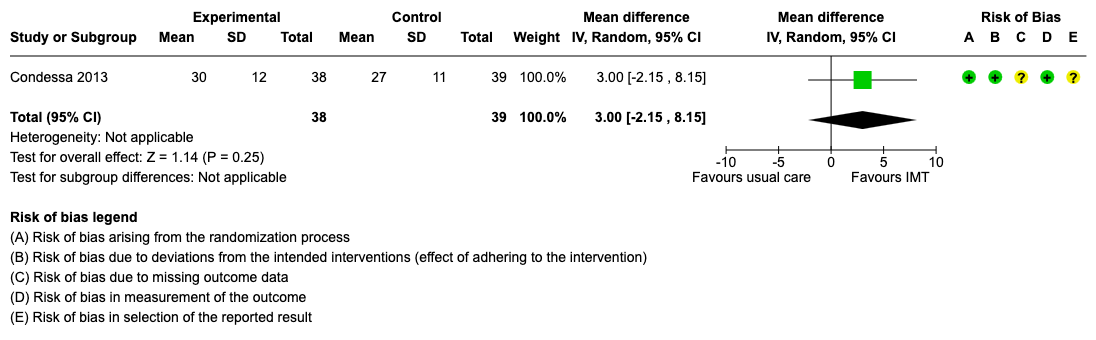
**

**eFigure 7**. Forest plot of the maximal expiratory pressure mean difference after completion of allocated treatment. IMT = inspiratory muscle training; SD = standard deviation; CI = confidence interval; + = low risk of bias; ? = some concerns for risk of bias.

**
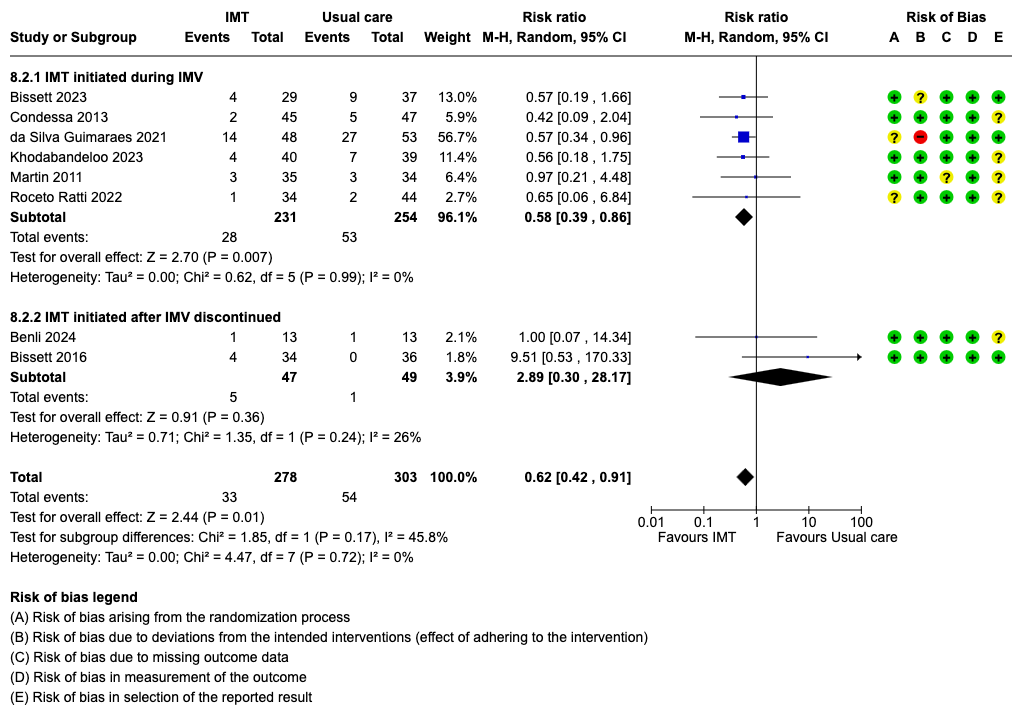
**

**eFigure 8**. Forest plot of the risk ratio of mortality by IMT initiation time. IMT = inspiratory muscle training; SD = standard deviation; CI = confidence interval; IMV = invasive mechanical ventilation; M-H = Mantel-Haenszel; + = low risk of bias; ? = some concerns for risk of bias; – = high risk of bias.

**
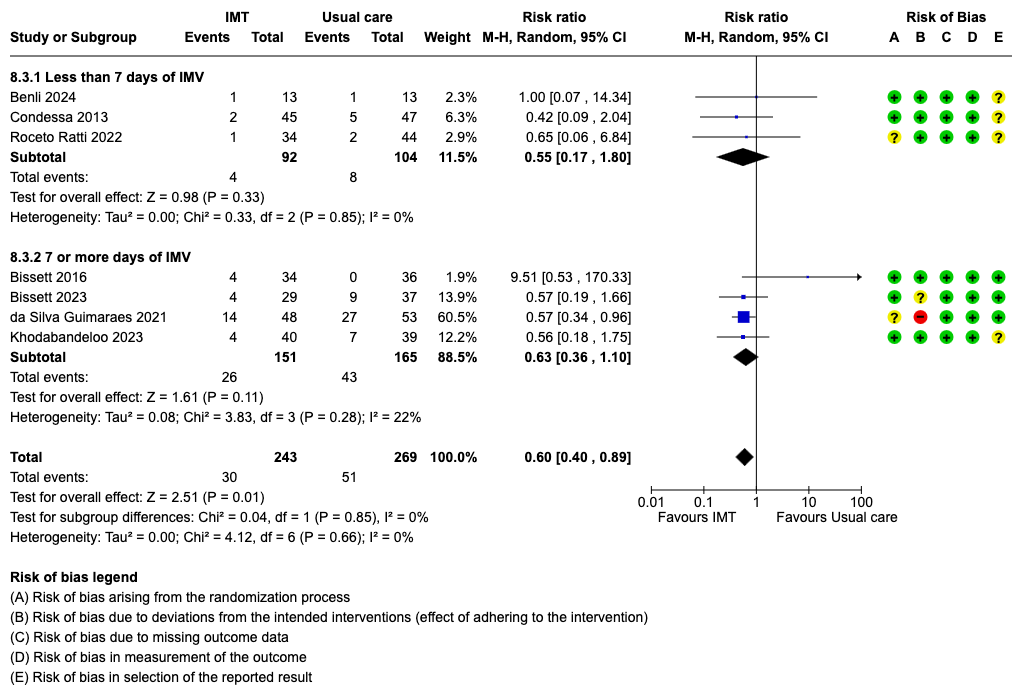
**

**eFigure 9**. Forest plot of the risk ratio of mortality by invasive mechanical ventilation duration. IMT = inspiratory muscle training; SD = standard deviation; CI = confidence interval; IMV = invasive mechanical ventilation; M-H = Mantel-Haenszel; + = low risk of bias; ? = some concerns for risk of bias; – = high risk of bias.

**
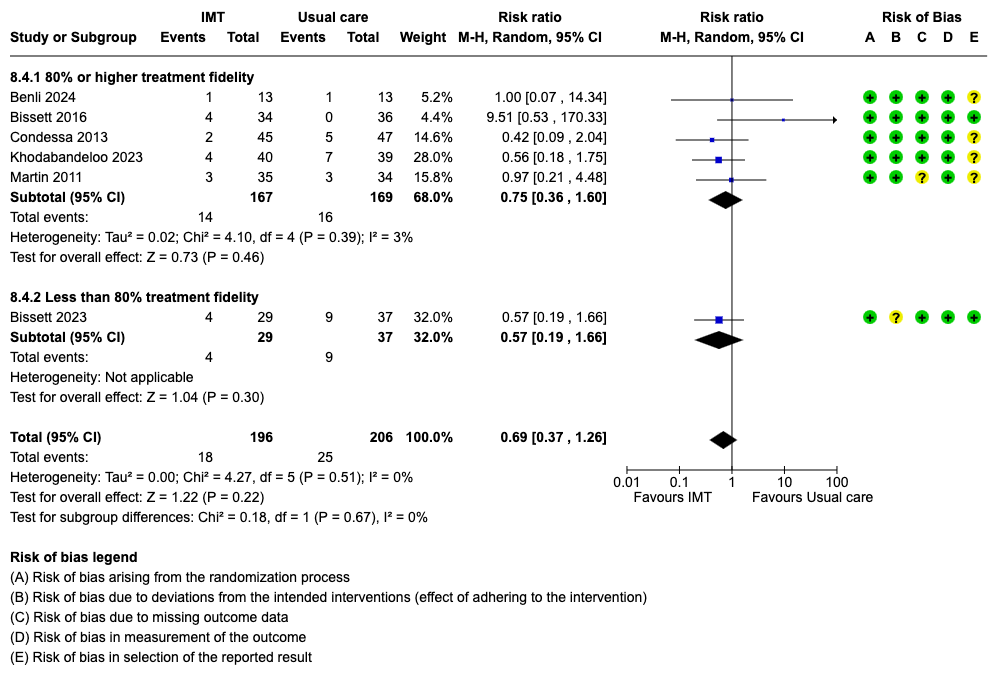
**

**eFigure 10**. Forest plot of the risk ratio of mortality by treatment fidelity. IMT = inspiratory muscle training; SD = standard deviation; CI = confidence interval; M-H = Mantel-Haenszel; + = low risk of bias; ? = some concerns for risk of bias.

**
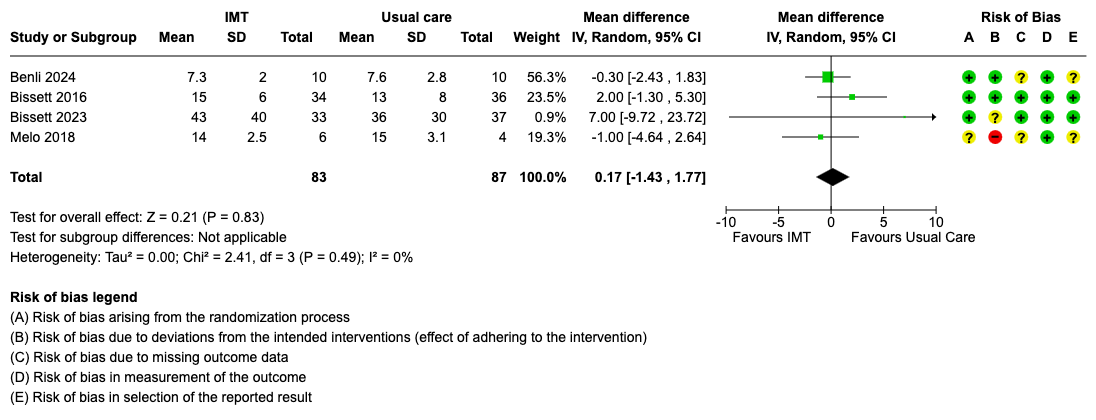
**

**eFigure 11**. Forest plot of the intensive care unit length of stay mean difference. IMT = inspiratory muscle training; SD = standard deviation; CI = confidence interval; + = low risk of bias; ? = some concerns for risk of bias; – = high risk of bias.

**
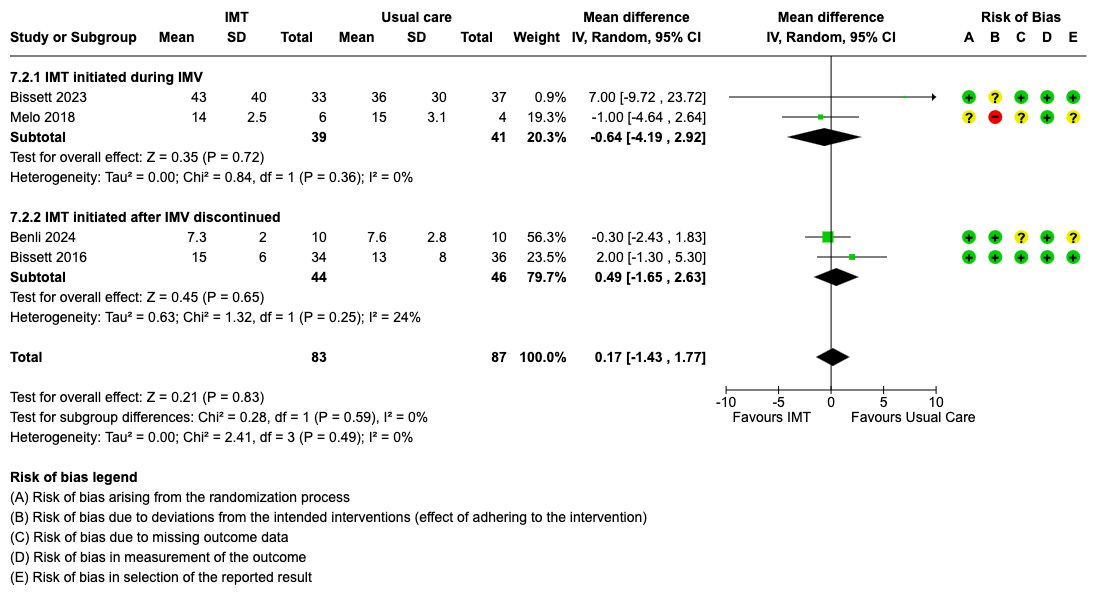
**

**eFigure 12**. Forest plot of the intensive care unit length of stay mean difference by IMT initiation time. IMT = inspiratory muscle training; SD = standard deviation; CI = confidence interval; IMV = invasive mechanical ventilation; + = low risk of bias; ? = some concerns for risk of bias; – = high risk of bias.

**
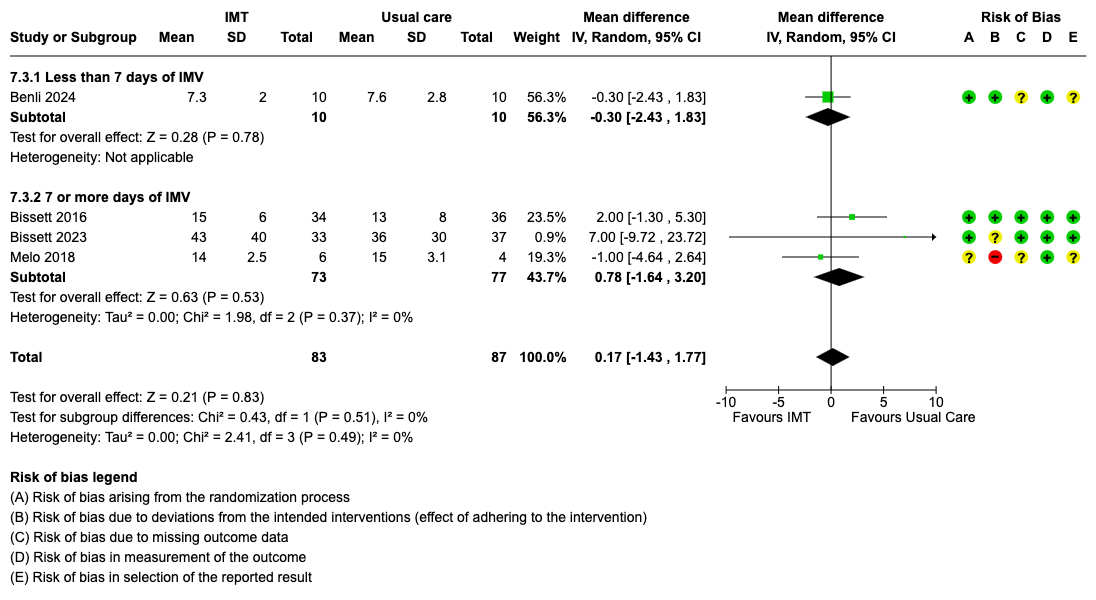
**

**eFigure 13**. Forest plot of the intensive care unit length of stay mean difference by invasive mechanical ventilation duration. IMT = inspiratory muscle training; SD = standard deviation; CI = confidence interval; IMV = invasive mechanical ventilation; + = low risk of bias; ? = some concerns for risk of bias; – = high risk of bias.

**
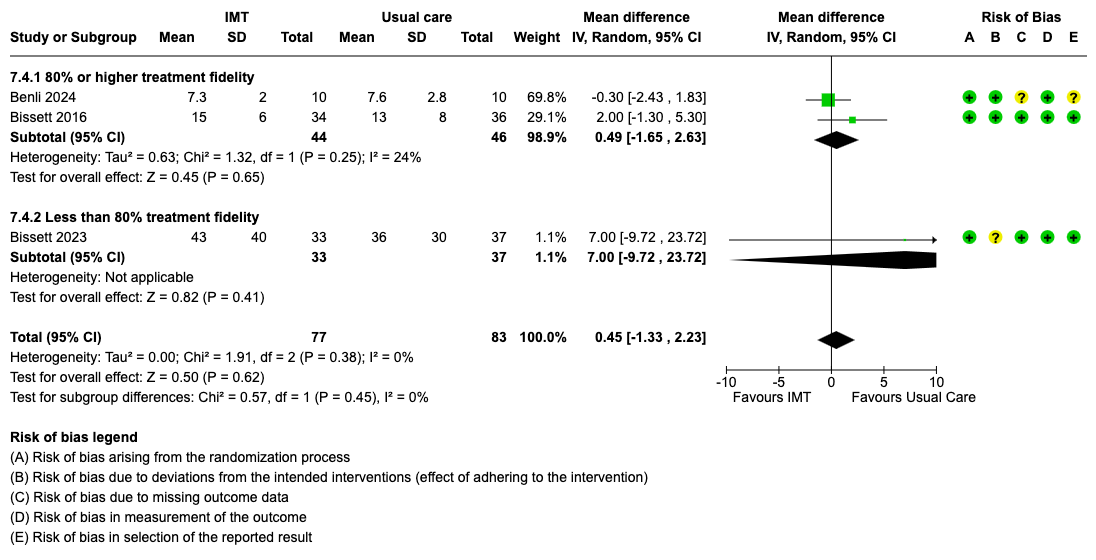
**

**eFigure 14**. Forest plot of the intensive care unit length of stay mean difference by treatment fidelity. IMT = inspiratory muscle training; SD = standard deviation; CI = confidence interval; IMV = invasive mechanical ventilation; + = low risk of bias; ? = some concerns for risk of bias.

**
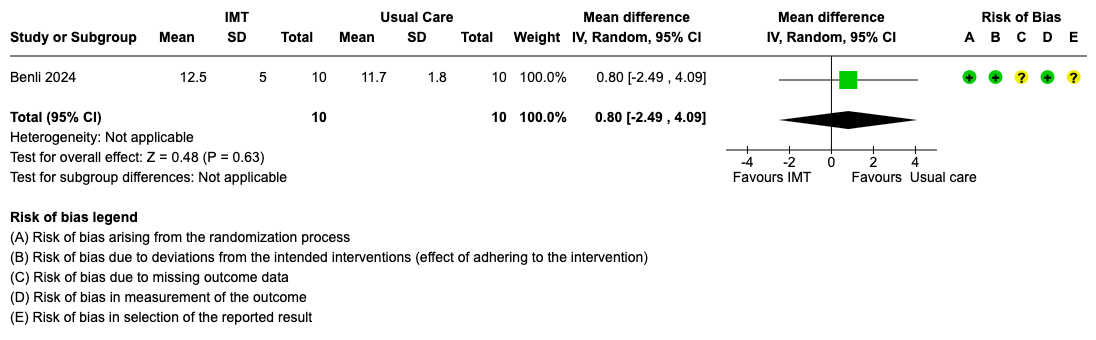
**

**eFigure 15**. Forest plot of the hospital length of stay mean difference. IMT = inspiratory muscle training; SD = standard deviation; CI = confidence interval; + = low risk of bias; ? = some concerns for risk of bias.

**
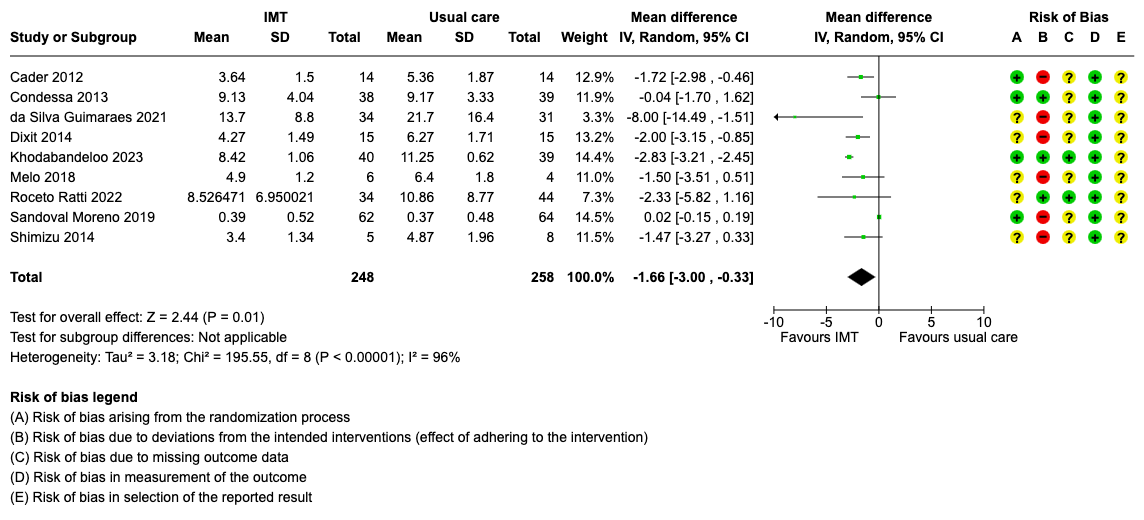
**

**eFigure 16**. Forest plot of the mean difference of invasive mechanical ventilator weaning time. IMT = inspiratory muscle training; SD = standard deviation; CI = confidence interval; + = low risk of bias; ? = some concerns for risk of bias; – = high risk of bias.

**
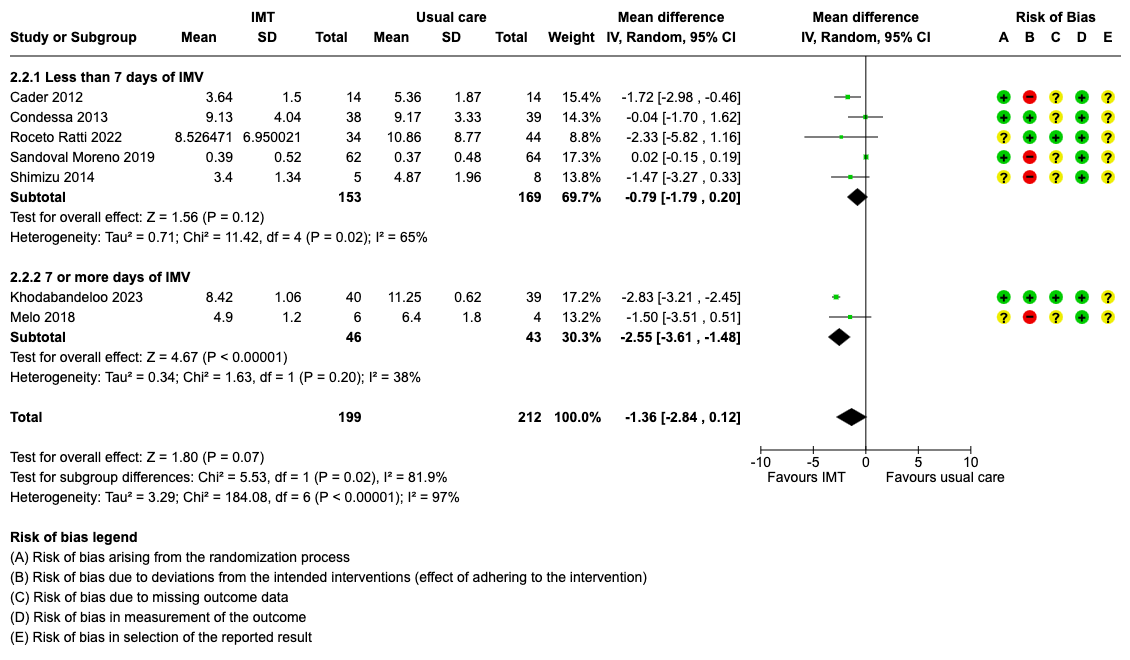
**

**eFigure 17**. Forest plot of the mean difference of invasive mechanical ventilator weaning time by invasive mechanical ventilation duration. IMT = inspiratory muscle training; SD = standard deviation; CI = confidence interval; IMV = invasive mechanical ventilation; + = low risk of bias; ? = some concerns for risk of bias; – = high risk of bias.

**
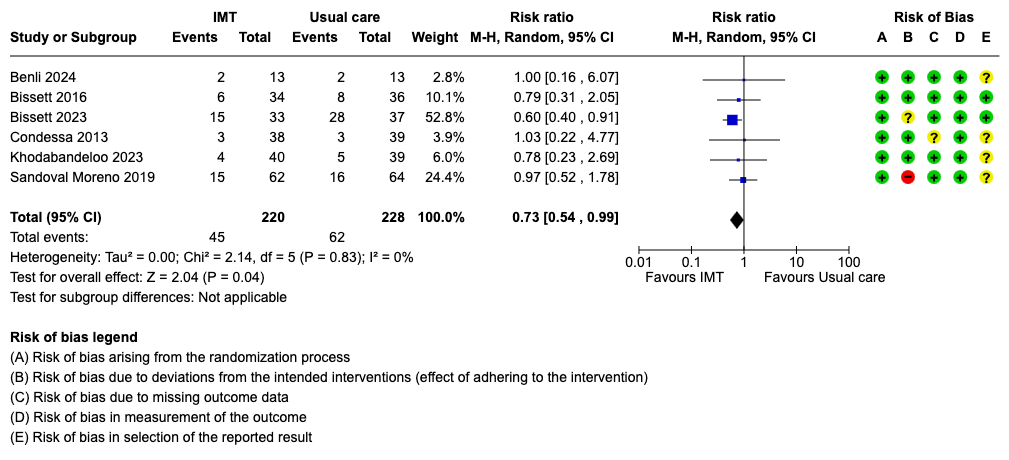
**

**eFigure 18**. Forest plot of the reintubation risk ratio. IMT = inspiratory muscle training; SD = standard deviation; CI = confidence interval; IMV = invasive mechanical ventilation; M-H = Mantel-Haenszel; + = low risk of bias; ? = some concerns for risk of bias; – = high risk of bias.

**
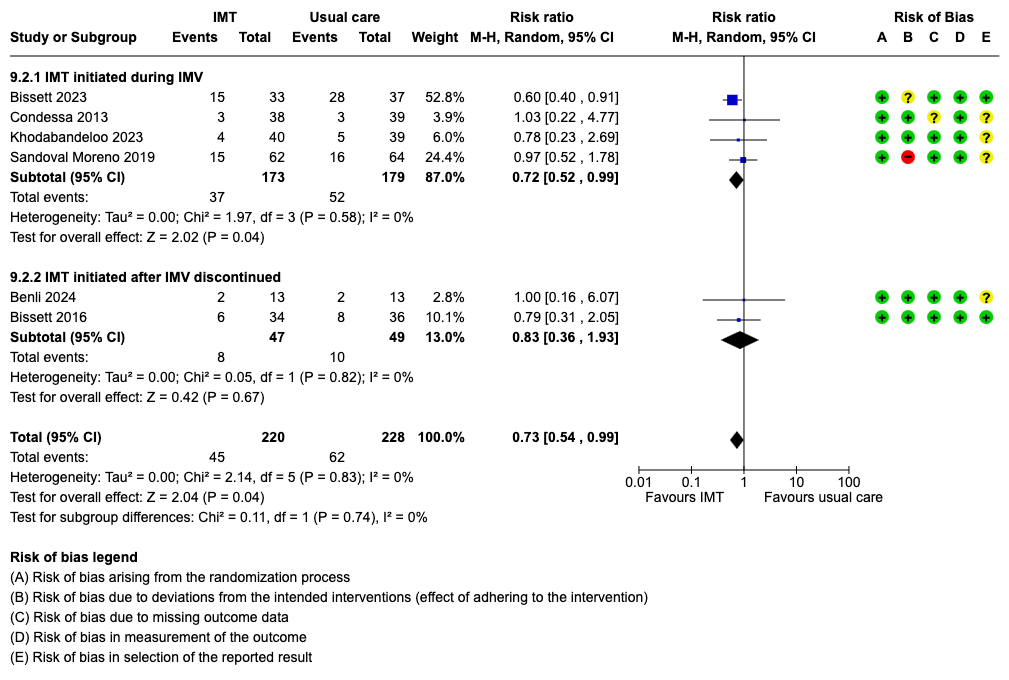
**

**eFigure 19**. Forest plot of the reintubation risk ratio by IMT initiation time. IMT = inspiratory muscle training; SD = standard deviation; CI = confidence interval; IMV = invasive mechanical ventilation; M-H = Mantel-Haenszel; + = low risk of bias; ? = some concerns for risk of bias; – = high risk of bias.

**
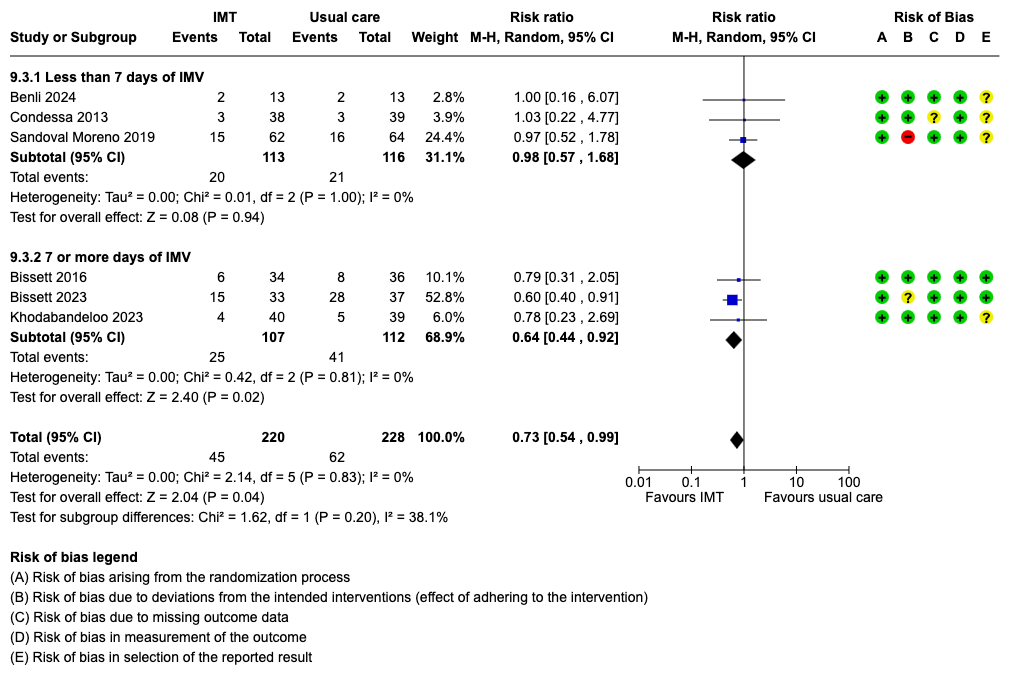
**

**eFigure 20**. Forest plot of the reintubation risk ratio by invasive mechanical ventilation duration. IMT = inspiratory muscle training; SD = standard deviation; CI = confidence interval; IMV = invasive mechanical ventilation; M-H = Mantel-Haenszel; + = low risk of bias; ? = some concerns for risk of bias; – = high risk of bias.

**
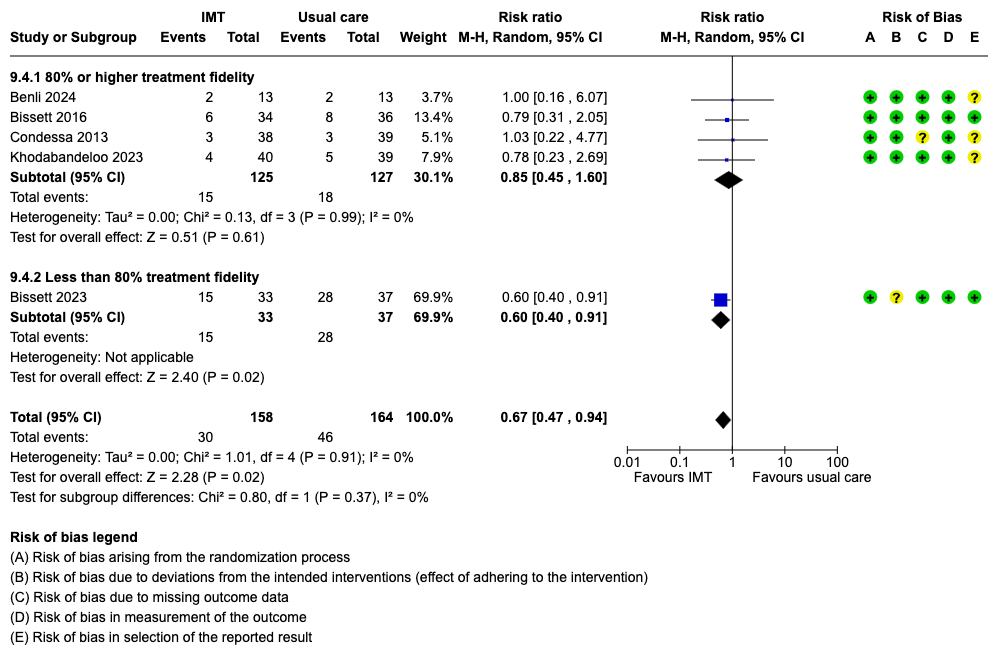
**

**eFigure 21**. Forest plot of the reintubation risk ratio by treatment fidelity. IMT = inspiratory muscle training; SD = standard deviation; CI = confidence interval; M-H = Mantel-Haenszel; + = low risk of bias; ? = some concerns for risk of bias.

**
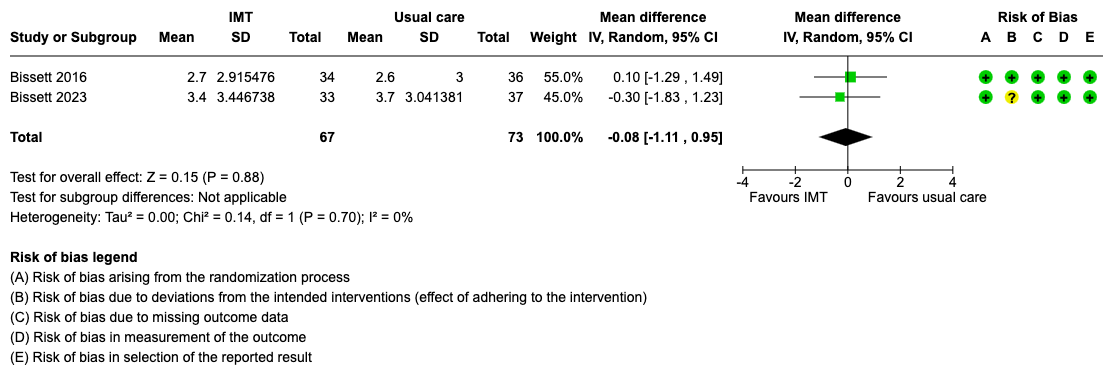
**

**eFigure 22**. Forest plot of the mean difference for dyspnea after completion of allocated treatment in the units of the Modified Borg Dyspnea Scale.[^48^](#_ENREF_48) IMT = inspiratory muscle training; SD = standard deviation; CI = confidence interval; IMV = invasive mechanical ventilation; + = low risk of bias; ? = some concerns for risk of bias; – = high risk of bias.

**
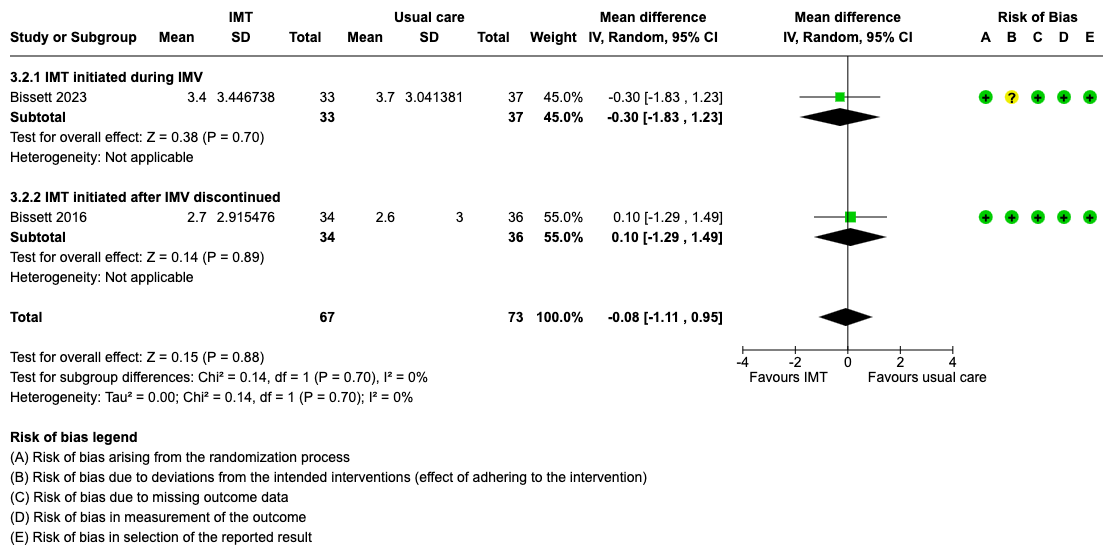
**

**eFigure 23**. Forest plot of the mean difference for dyspnea after completion of allocated treatment in the units of the Modified Borg Dyspnea Scale[^48^](#_ENREF_48) by IMT initiation time. IMT = inspiratory muscle training; SD = standard deviation; CI = confidence interval; IMV = invasive mechanical ventilation; + = low risk of bias; ? = some concerns for risk of bias; – = high risk of bias.

**
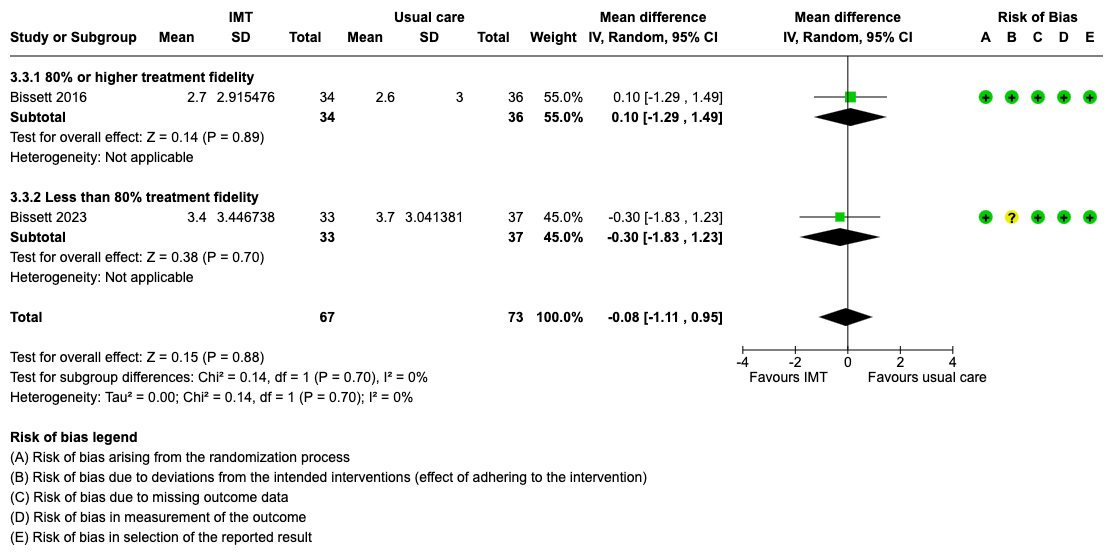
**

**eFigure 24**. Forest plot of the mean difference for dyspnea after completion of allocated treatment in the units of the Modified Borg Dyspnea Scale[^48^](#_ENREF_48) by treatment fidelity. IMT = inspiratory muscle training; SD = standard deviation; CI = confidence interval; + = low risk of bias; ? = some concerns for risk of bias; – = high risk of bias.

**
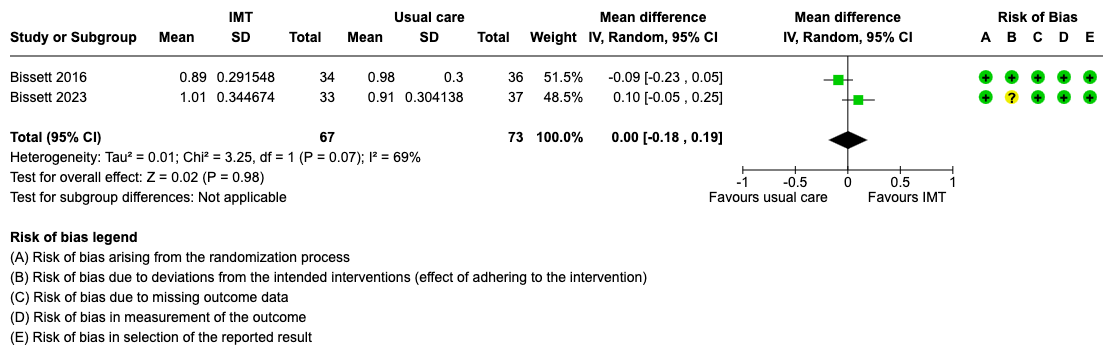
**

**eFigure 25**. Forest plot of the mean difference for respiratory endurance after completion of allocated treatment in the units of the Fatigue Resistance Index.[^49^](#_ENREF_49) IMT = inspiratory muscle training; SD = standard deviation; CI = confidence interval; + = low risk of bias; ? = some concerns for risk of bias.

**
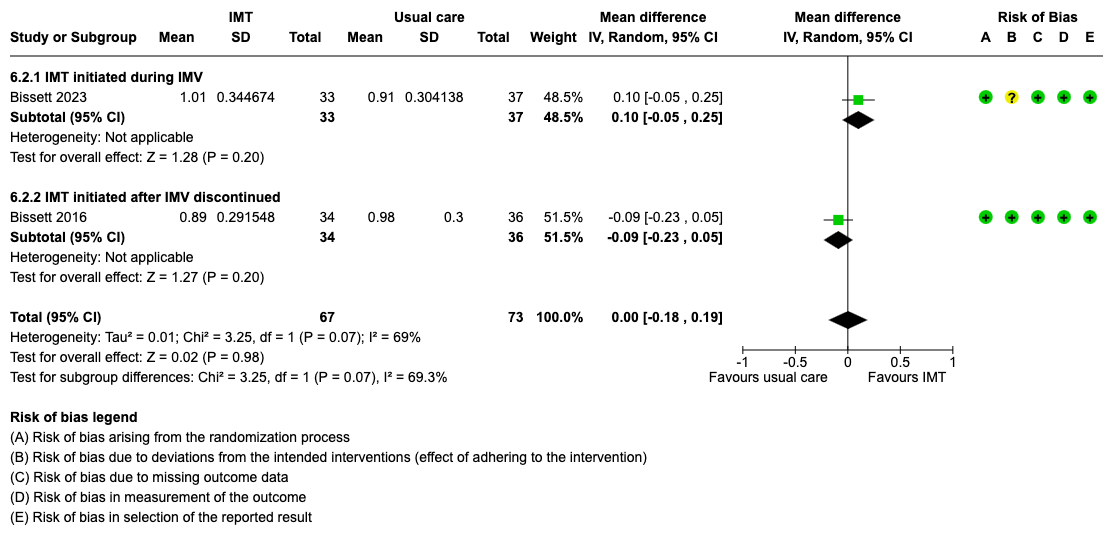
**

**eFigure 26**. Forest plot of the mean difference for respiratory endurance after completion of allocated treatment in the units of the Fatigue Resistance Index[^49^](#_ENREF_49) by IMT initiation time. IMT = inspiratory muscle training; SD = standard deviation; CI = confidence interval; IMV = invasive mechanical ventilation; + = low risk of bias; ? = some concerns for risk of bias.

**
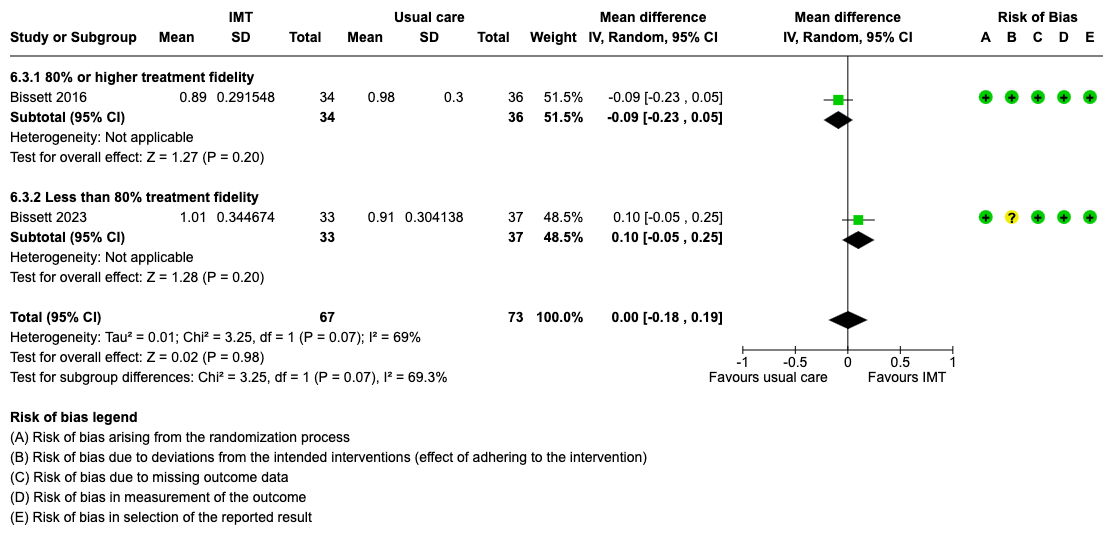
**

**eFigure 27**. Forest plot of the mean difference for respiratory endurance after completion of allocated treatment in the units of the Fatigue Resistance Index[^49^](#_ENREF_49) by treatment fidelity. IMT = inspiratory muscle training; SD = standard deviation; CI = confidence interval; + = low risk of bias; ? = some concerns for risk of bias.

**
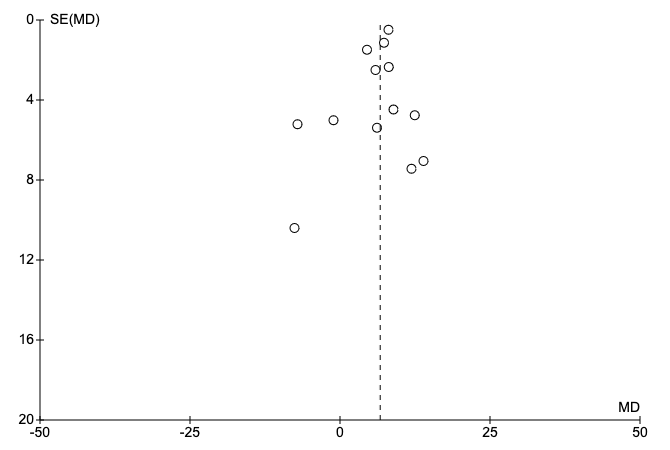
**

**eFigure 28**. Funnel plots for maximal inspiratory pressure.

**References**

1. Glanville J, Kotas E, Featherstone R, Dooley G. Which are the most sensitive search filters to identify randomized controlled trials in MEDLINE? *J Med Libr Assoc*. Oct 1 2020;108(4):556-563. doi:10.5195/jmla.2020.912

2. Glanville J, Foxlee R, Wisniewski S, Noel-Storr A, Edwards M, Dooley G. Translating the Cochrane EMBASE RCT filter from the Ovid interface to Embase.com: a case study. *Health Info Libr J*. Sep 2019;36(3):264-277. doi:10.1111/hir.12269

3. Glanville J, Dooley G, Wisniewski S, Foxlee R, Noel-Storr A. Development of a search filter to identify reports of controlled clinical trials within CINAHL Plus. *Health Info Libr J*. Mar 2019;36(1):73-90. doi:10.1111/hir.12251

4. Aldrich TK, Karpel JP, Uhrlass RM, Sparapani MA, Eramo D, Ferranti R. Weaning from mechanical ventilation: adjunctive use of inspiratory muscle resistive training. *Critical care medicine*. 1989;17(2):143-7.

5. Bento H, Fisk E, Johnson E, et al. Inspiratory Muscle Training While Hospitalized With Acute COVID-19 Respiratory Failure: a Randomized Controlled Trial. 2023;14(3):134‐142. doi:10.1097/JAT.0000000000000217

6. Caruso P, Denari SD, Ruiz SA, Bernal KG, Manfrin GM, Friedrich C, Deheinzelin D. Inspiratory muscle training is ineffective in mechanically ventilated critically ill patients. 2005;60(6):479‐484. doi:10.1590/s1807-59322005000600009

7. Chang H-Y, Hsiao H-C, Chang H-L. Impact of Inspiratory Muscle Training on Weaning Parameters in Prolonged Ventilator-Dependent Patients: A Preliminary Study. *SAGE Open Nursing*. 2022;8:1-10. doi:10.1177/23779608221111717

8. Dellweg D, Reissig K, Hoehn E, Siemon K, Haidl P. Inspiratory muscle training during rehabilitation in successfully weaned hypercapnic patients with COPD. 2017;123:116‐123. doi:10.1016/j.rmed.2016.12.006

9. Elbouhy MS, AbdelHalim HA, Hashem AMA. Effect of respiratory muscles training in weaning of mechanically ventilated COPD patients. 2014;63(3):679‐687. doi:10.1016/j.ejcdt.2014.03.008

10. Ferreira A, Tonella R, Ratti L, et al. Is there difference between two inspiratory muscle training in elderly in prolonged weaning?-Pilot study. 2019;54doi:10.1183/13993003.congress-2019.PA1152

11. Koch R, Augusto TRL, Ramos AG, Müller PT. Inspiratory Muscle Training Potentiates the Beneficial Effects of Proportional Assisted Ventilation on Exertional Dyspnea and Exercise Tolerance in COPD: a Proof-of-Concept Randomized and Controlled Trial. 2020;17(4):384‐391. doi:10.1080/15412555.2020.1789085

12. Reginault T, Martinez Alejos R, Jean-Francois B, Roxane C, Eric F, Frederic V. Comparison of the impact of three inspiratory muscle training programs on diaphragm strength and endurance in intubated and mechanically ventilated patients in difficult weaning: a multicentric controlled randomized parallel trial. 2023;11doi:10.1186/s40635-023-00546-y

13. Benli RK, Yurdalan U, Yilmaz B, Adiguzel N. Effect of post-extubation inspiratory muscle training on diaphragmatic function in mechanically ventilated patients: a randomized controlled trial. 2024;doi:10.17219/acem/174815

14. Bissett B, Leditschke IA, Neeman T, Boots R, Paratz J. Inspiratory Muscle Training to Enhance Recovery from Prolonged Mechanical Ventilation: A Randomized Trial. 2016;193(Meeting Abstracts):A2613.

15. Bissett BM, Leditschke A, Neeman T, Boots R, Paratz J. Inspiratory muscle training to enhance recovery from mechanical ventilation: A randomised trial. *Thorax*. 2016;71(9):812-9.

16. Bissett B, Wang J, Neeman T, Leditschke A, Boots R, Paratz J. Which patients benefit most from inspiratory muscle training in ICU? Secondary analysis of a randomised controlled trial. *Australian Critical Care*. 2018;31(2):131-131. doi:10.1016/j.aucc.2017.12.053

17. Bissett BM, Leditschke IA, Neeman T, et al. Does mechanical threshold inspiratory muscle training promote recovery and improve outcomes in patients who are ventilator-dependent in the intensive care unit? The IMPROVE randomised trial. 2023;36(4):613‐621. doi:10.1016/j.aucc.2022.07.002

18. Cader SA, de Souza Vale RG, Zamora VE, Costa CH, Dantas EH. Extubation process in bed-ridden elderly intensive care patients receiving inspiratory muscle training: a randomized clinical trial. 2012;7:437‐443. doi:10.2147/CIA.S36937

19. Cader SA, Vale RG, Castro JC, et al. Inspiratory muscle training improves maximal inspiratory pressure and may assist weaning in older intubated patients: a randomised trial. 2010;56(3):171-7.

20. Inspiratory Muscle Training in Weaning. <https://clinicaltrials.gov/study/NCT00922493>

21. Condessa RL, Brauner JS, Saul AL, Baptista M, Silva AC, Vieira SR. Inspiratory muscle training did not accelerate weaning from mechanical ventilation but did improve tidal volume and maximal respiratory pressures: a randomised trial. 2013;59(2):101‐107. doi:10.1016/S1836-9553(13)70162-0

22. da Silva Guimarães B, de Souza LC, Cordeiro HF, et al. Inspiratory Muscle Training With an Electronic Resistive Loading Device Improves Prolonged Weaning Outcomes in a Randomized Controlled Trial. 2021;49(4):589‐597. doi:10.1097/CCM.0000000000004787

23. Souza L, Guimaraes B, Lugon J. Use of a new isokinetic device oriented by software for inspiratory muscle training in prolonged weaning. 2017;50doi:10.1183/1393003.congress-2017.PA1864

24. Lugon JR. Use of a New Isokinetic Device for Inspiratory Muscle Training. <https://clinicaltrials.gov/study/NCT02932189>

25. Guimaraes BL, De souza L, Guimaraes F, et al. Use of a new isokinetic device oriented by software for inspiratory muscle training in prolonged weaning. 2017;21(2)doi:10.1186/s13054-017-1706-1

26. Dixit A, Prakash S. Effects of Threshold Inspiratory Muscle Training Versus Conventional Physiotherapy on the Weaning Period of Mechanically Ventilated Patients: A Comparative Study. 2014:

27. dos Santos Pascotini F, Denardi C, Nunes GO, Trvisan ME, da Pieve Antunes V. Treinamento muscular respiratório em pacientes em desmame da ventilação mecânica. *ABCS health sciences*. 2014;39(1)

28. Khodabandeloo F, Froutan R, Yazdi AP, Shakeri MT, Mazlom SR, Moghaddam AB. The effect of threshold inspiratory muscle training on the duration of weaning in intensive care unit-admitted patients: a randomized clinical trial. 2023;28:44. doi:10.4103/jrms.jrms_757_22

29. Comparison of the effect of inspiratory muscle training with common chest physiotherapy on the weaning duration in patients hospitalized in intensive care units. <https://www.cochranelibrary.com/central/doi/10.1002/central/CN-02279850/full>

30. Martin AD, Smith BK, Davenport PD, et al. Inspiratory muscle strength training improves weaning outcome in failure to wean patients: a randomized trial. 2011;15(2):R84. doi:10.1186/cc10081

31. Martin A, Caruso L, Banner M, Layon A, Gabrielli A. Inspiratory muscle strength training improves weaning outcome in failure to wean patients. *Critical Care Medicine*. 2009;37(12 SUPPL.):A34. doi:<https://dx.doi.org/10.1097/01.ccm.0000365439.11849.a2>

32. Inspiratory Muscle Training in Ventilator Dependent Patients. <https://clinicaltrials.gov/study/NCT00419458>

33. Melo P, Pereira D, Maldaner V, et al. Inspiratory muscular traning in non-collaborative patients using mechanical ventilation in the ICU. 2018;6(Supplement 2) (no pagination)doi:10.1186/s40635-018-0201-6

34. Melo PF, Da Silva V, Vieira L, et al. High intensity inspiratory muscle training in patients with traumatic brain injury under mechanical ventilation: preliminary results of a randomized controlled trial. 2017;195doi:10.1164/ajrccm-conference.2017.A104

35. Mohamed AR, El Basiouny HM, Salem NM. Response of mechanically ventilated respiratory failure patients to respiratory muscles training. *Med J Cairo Univ*. 2014;82:19-24.

36. Ibrahiem AAA, Mohamed AR, Elbasiouny HS. Effect of respiratory muscles training in addition to standard chest physiotherapy on mechanically ventilated patients. *J Med Res Pract*. 2014;3:52-8.

37. Roceto Ratti LDS, Marques Tonella R, Castilho de Figueir do L, Bredda Saad IA, Eiras Falcão AL, Martins de Oliveira PP. Inspiratory Muscle Training Strategies in Tracheostomized Critically Ill Individuals. 2022;67(8):939‐948. doi:10.4187/respcare.08733

38. Tonella RM, Ratti L, Delazari LEB, et al. Inspiratory Muscle Training in the Intensive Care Unit: A New Perspective. *J Clin Med Res*. Nov 2017;9(11):929-934. doi:10.14740/jocmr3169w

39. Ratti L, Tonella R, Figueiredo L, Saad I, Falcao A, Martins PP. Different loads of inspiratory muscle training in mechanical ventilation weaning: randomized trial. 2018;52doi:10.1183/13993003.congress-2018.PA2328

40. Saad IAB, Tonella R, Roceto LS, Delazari LEB, Castilho L, Falcao ALE, Silva PS. A new device for inspiratory muscle training in patients with tracheostomy tube in ICU: a randomized trial. 2014;44

41. Sandoval Moreno LM, Casas Quiroga IC, Wilches Luna EC, García AF. Efficacy of respiratory muscle training in weaning of mechanical ventilation in patients with mechanical ventilation for 48hours or more: a Randomized Controlled Clinical Trial. 2019;43(2):79‐89. doi:10.1016/j.medin.2017.11.010

42. Effects of Respiratory Muscle Training in Mechanically Ventilated Adults. <https://www.cochranelibrary.com/central/doi/10.1002/central/CN-02039693/full>

43. Shimizu JM, Manzano RM, Quitério RJ, Alegria VTdC, Junqueira TT, El-Fakhouri S, Ambrozin ARP. Determinant factors for mortality of patients receiving mechanical ventilation and effects of a protocol muscle training in weaning. *Manual Therapy, Posturology & Rehabilitation Journal*. 06/02 2014;0(0):1-7.

44. Shrestha BK, Qutob HF, Berry M, et al. Feasibility and safety of inspiratory muscle training in critically ill intubated patient. 2014;189(no pagination)

45. Van Hollebeke M, Louvaris Z, Clerckx B, Muller J, Gosselink R, Hermans G, Langer D. Evolution of inspiratory muscle training in patients with weaning difficulties. 2019;54doi:10.1183/13993003.congress-2019.PA2202

46. Van Hollebeke M, Muelas L, Barbosa MH, Clerckx B, Hermans G, Langer D, Gosselink R. Inspiratory muscle training with tapered flow resistive loading versus mechanical threshold loading in difficult to wean patients. 2020;56doi:10.1183/13993003.congress-2020.3028

47. Van Hollebeke M, Poddighe D, Clerckx B, et al. High-Intensity Inspiratory Muscle Training Improves Scalene and Sternocleidomastoid Muscle Oxygenation Parameters in Patients With Weaning Difficulties: a Randomized Controlled Trial. 2022;13:786575. doi:10.3389/fphys.2022.786575

48. Borg GA. Psychophysical bases of perceived exertion. *Med Sci Sports Exerc*. 1982 1982;14(5):377-381.

49. Clanton T, Carverly PM, Celli BR. Tests of respiratory muscle endurance. Review. *American Journal of Respiratory and Critical Care Medicine*. 2002;166(4):559-570.
